# Live Attenuated Tularemia Vaccines for Protection Against Respiratory Challenge With Virulent *F. tularensis* subsp. *tularensis*

**DOI:** 10.3389/fcimb.2018.00154

**Published:** 2018-05-15

**Authors:** Qingmei Jia, Marcus A. Horwitz

**Affiliations:** Division of Infectious Diseases, Department of Medicine, 37-121 Center for Health Sciences, School of Medicine, University of California, Los Angeles, Los Angeles, CA, United States

**Keywords:** tularemia, *Francisella tularensis*, tularemia vaccine, live attenuated vaccine, bioterrorism, homologous vaccine, heterologous vaccine, prime-boost vaccine

## Abstract

*Francisella tularensis* is the causative agent of tularemia and a Tier I bioterrorism agent. In the 1900s, several vaccines were developed against tularemia including the killed “Foshay” vaccine, subunit vaccines comprising *F. tularensis* protein(s) or lipoproteins(s) in an adjuvant formulation, and the *F. tularensis* Live Vaccine Strain (LVS); none were licensed in the U.S.A. or European Union. The LVS vaccine retains toxicity in humans and animals—especially mice—but has demonstrated efficacy in humans, and thus serves as the current gold standard for vaccine efficacy studies. The U.S.A. 2001 anthrax bioterrorism attack spawned renewed interest in vaccines against potential biowarfare agents including *F. tularensis*. Since live attenuated—but not killed or subunit—vaccines have shown promising efficacy and since vaccine efficacy against respiratory challenge with less virulent subspecies *holarctica* or *F. novicida*, or against non-respiratory challenge with virulent subsp. *tularensis* (Type A) does not reliably predict vaccine efficacy against respiratory challenge with virulent subsp. *tularensis*, the route of transmission and species of greatest concern in a bioterrorist attack, in this review, we focus on live attenuated tularemia vaccine candidates tested against respiratory challenge with virulent Type A strains, including homologous vaccines derived from mutants of subsp. *holarctica, F. novicida*, and subsp. *tularensis*, and heterologous vaccines developed using viral or bacterial vectors to express *F. tularensis* immunoprotective antigens. We compare the virulence and efficacy of these vaccine candidates with that of LVS and discuss factors that can significantly impact the development and evaluation of live attenuated tularemia vaccines. Several vaccines meet what we would consider the minimum criteria for vaccines to go forward into clinical development—safety greater than LVS and efficacy at least as great as LVS, and of these, several meet the higher standard of having efficacy ≥LVS in the demanding mouse model of tularemia. These latter include LVS with deletions in *purMCD, sodB*_*Ft*_, *capB* or *wzy*; LVS Δ*capB* that also overexpresses Type VI Secretion System (T6SS) proteins; FSC200 with a deletion in *clpB*; the single deletional *purMCD* mutant of *F. tularensis* SCHU S4, and a heterologous prime-boost vaccine comprising LVS Δ*capB* and *Listeria monocytogenes* expressing T6SS proteins.

## Introduction

*Francisella tularensis*, the causative agent of tularemia, was originally named *Bacterium tularense* by McCoy and Chapin in 1911, who discovered it as the causative agent of a “plague like disease” prevalent among the ground squirrels in Tulare County, California (McCoy GW, 1912; Francis, 1925). The bacterium was designated as *Pasteurella tularensis* in 1920's and later renamed *Francisella tularensis* in honor of the contributions made by Dr. Edward Francis among others (Francis, 1928; Weinberg, 2004; Sjostedt, 2007). Several vaccine strategies were developed in the 1900s against virulent *F. tularensis* infection, including killed whole cell vaccines, subunit vaccines, and live attenuated homologous vaccines (Wayne Conlan and Oyston, 2007). A killed whole cell vaccine, the earliest vaccine developed against tularemia and referred to as the “Foshay” vaccine (Foshay, 1932; Foshay et al., 1942), was prepared from phenolized liquid culture and was not highly protective against subsequent systemic and aerosol challenge with highly virulent strains of *F. tularensis* in mice, guinea pigs, rabbits, and humans (Foshay, 1932; Foshay et al., 1942; Kadull et al., 1950; Vanmetre and Kadull, 1959; Eigelsbach and Downs, 1961; Saslaw et al., 1961a,b). Subunit vaccines comprising *F. tularensis* protein(s) or lipoproteins(s) in an adjuvant formulation also failed to demonstrate strong protective immunity against virulent non-Type A or Type A *F. tularensis* in animal models (Sjostedt et al., 1992b; Golovliov et al., 1995; Fulop et al., 2001; Conlan et al., 2002). Live attenuated homologous vaccines showed greater promise. The earliest live attenuated homologous tularemia vaccine, Live Vaccine Strain or LVS, derived from a virulent isolate of *F. tularensis* subsp. *holarctica* (Type B), was developed by U.S.A. and Russian scientists in the 1950s (Eigelsbach and Downs, 1961). However, LVS retained considerable virulence in animals and provided incomplete protection to humans challenged with *F. tularensis* subsp. *tularensis* (Type A) by aerosol, the route of transmission of greatest concern in a bioterrorist attack (Saslaw et al., 1961a; Hornick and Eigelsbach, 1966; Fortier et al., 1991). The fundamental mechanism of LVS attenuation is not fully understood, although *pilA* and FTT0918 have been identified as contributing to LVS virulence (Salomonsson et al., 2009). The LVS vaccine has not been licensed in the U.S.A.^1^^,^^2^ or in the European Union^3^.

Until 2001, few researchers were actively working on vaccine development against tularemia. However, in the wake of the September 11, 2001 terrorist attack on the World Trade Center and the U.S.A. anthrax bioterrorism attack 1 week later, there has been renewed interest in vaccine development against tularemia and other potential biowarfare agents. This has been accompanied by a substantial increase in publications on development of tularemia vaccines, mostly on live attenuated vaccine candidates, including defined mutants of *F. tularensis* subsp. *holarctica* (Bakshi et al., 2006, 2008; Pechous et al., 2006, 2008; Li et al., 2007; Sebastian et al., 2007, 2009; Meibom et al., 2008; Sammons-Jackson et al., 2008; Santiago et al., 2009; Jia et al., 2010; Zarrella et al., 2011; Kim et al., 2012; Schmitt et al., 2012; Barrigan et al., 2013; Golovliov et al., 2013; Mahawar et al., 2013; Straskova et al., 2015; Suresh et al., 2015), *F. novicida* (Pammit et al., 2006; Tempel et al., 2006; Mohapatra et al., 2007; Quarry et al., 2007; Kanistanon et al., 2008; West et al., 2008; Cong et al., 2009; Sanapala et al., 2012; Signarovitz et al., 2012; Chu et al., 2014; Cunningham et al., 2014), and subsp. *tularensis* (Twine et al., 2005, 2012; Qin et al., 2008, 2009; Conlan et al., 2010; Michell et al., 2010; Shen et al., 2010; Ireland et al., 2011; Rockx-Brouwer et al., 2012; Reed et al., 2014; Santiago et al., 2015), but also on live attenuated heterologous vaccines expressing *F. tularensis* proteins (Jia et al., 2009; Kaur et al., 2012; Banik et al., 2015) and recombinant LVS vaccines expressing *F. tularensis* proteins (Jia et al., 2013, 2016). Some of these vaccine candidates have been reviewed elsewhere (Conlan, 2011; Marohn and Barry, 2013; Elkins et al., 2016). Because vaccine efficacy against respiratory challenge with subsp. *holarctica* or against non-respiratory challenge with subsp. *tularensis* does not reliably predict vaccine efficacy against respiratory challenge with subsp. *tularensis* (Conlan, 2011; Marohn and Barry, 2013), as noted the route of transmission and species of greatest concern in a bioterrorist attack, in this review, we focus on live attenuated vaccines that have been tested against respiratory challenge with virulent *F. tularensis* subsp. *tularensis*—the SCHU S4 strain originally isolated from a human ulcer in 1941 (Eigelsbach et al., 1951) or FSC033 (*Francisella* Strain Collection from Swedish Defense Research Agency, Sweden) originally isolated from a squirrel in Georgia (Forsman et al., 1994).

## Tularemia and *F. tularensis*

Tularemia occurs in nature as a fatal bacteremia of various rodents and other animals, such as rabbits, and is transmitted to humans as an accidental infection (Francis, 1925). Depending primarily on the route of transmission, there are several clinical forms of tularemia, including ulceroglandular and glandular tularemia caused by an arthropod bite or skin contact with an infected animal; oculoglandular tularemia caused by direct infection of the eye; oropharyngeal tularemia caused by ingestion of water contaminated by infected rodents or other animals or consumption of under-cooked meat from an infected animal; typhoidal tularemia from any mode of transmission; and pneumonic tularemia caused by inhalation of aerosolized bacteria. Typhoidal and pneumonic tularemia are the most dangerous forms as they carry a high fatality rate−30–60% if untreated (Dennis et al., 2001; Matyas et al., 2007). Because *F. tularensis* is one of the most pathogenic human pathogens known and can be spread by aerosol transmission to cause highly fatal pneumonic tularemia, it is classified as a Tier 1 select agent of bioterrorism, i.e., among the most likely pathogens to be deliberately used in a bioterrorist attack. In fact, Japan, the U.S.A., and the U.S.S.R. have stockpiled *F. tularensis* as a bioweapon in the past (Harris, 1992; Christopher et al., 1997; Alibek, 1999). *F. tularensis* subsp. *tularensis* (Type A), prevalent in North America, is the most virulent subsp.; intracutaneous challenge or inhalation of as few as 10–50 colony forming units (CFU) (SCHU S4 strain) is able to cause clinical tularemia in humans (McCrumb, 1961; Saslaw et al., 1961a,b). *F. tularensis* subsp. *holarctica* (Type B), found in Europe, Asia and North America, is less virulent than subsp. *tularensis*. Subsp. *mediasiatica*, found in the Central Asia and the former USSR, is of similar virulence to subsp. *holarctica* (Sjostedt, 2007). *F. novicida*, genetically closely related to *F. tularensis* and frequently referred to as subsp. *novicida* (Rohmer et al., 2007; Huber et al., 2010), is currently classified as a separate species (Johansson et al., 2010; Kingry and Petersen, 2014). *F. novicida* shows low virulence in experimental models, occasionally causes disease in immunocompromised individuals, and has been isolated from patients with various clinical entities in the U.S.A., Canada, Australia, and Spain (Bernard et al., 1994; WHO, 2007).

## Biology and pathogenesis of experimental tularemia and the host response

*F. tularensis* is a facultative intracellular pathogen that is capable of infecting multiple types of eukaryotic cells, including macrophages. Using a human macrophage-like cell line (THP-1), Clemens et al. showed that *F. tularensis* subsp. *tularensis* SCHU S4 and subsp. *holarctica* LVS enter human macrophages via a unique complement-dependent process termed looping phagocytosis; then enter a unique fibrillar-coated phagosome; and finally exit the phagosome to multiply freely in the cytoplasm using a Type VI Secretion System (T6SS) (Clemens et al., 2004, 2005, 2015; Clemens and Horwitz, 2007). In addition to the macrophage, *F. tularensis* can infect alveolar type II epithelial cells, neutrophils, dendritic cells, and others (Metzger et al., 2007). Studies on the pathogenesis of experimental tularemia were mostly conducted in mice and to a lesser extent in Fisher rats and non-human primates (Jemski, 1981; Lyons and Wu, 2007; Hutt et al., 2017). Upon i.d. or aerosol infection, *F. tularensis* quickly disseminates systemically to spleen, liver, lung, lymph nodes, and bone marrow, and multiplies in these organs (Conlan et al., 2003; Fritz et al., 2014). As with many other intracellular pathogens, *F. tularensis* does not produce a toxin to cause tissue damage. Instead, *F. tularensis* damages tissues by invasion and destruction. Multiplication of *F. tularensis*, especially subsp. *tularensis*, in the tissues fails to trigger—but rather actively suppresses—innate immune responses in humans, which likely contributes to its enhanced pathogenicity (Bosio et al., 2007; Gillette et al., 2014). Although various immune responses to LVS vaccination and *F. tularensis* infection have been identified in animal models, correlates of protection are still not known. Both LVS vaccination and subsp. *tularensis* infection induce antibody responses. In the mouse, LVS vaccination induces *F. tularensis* antigen specific humoral (antibodies and B cells) and cellular (both CD4+ and CD8+ T cells) immune responses. While T-cell mediated immune responses are important for control of primary *Francisella* infection or vaccine-induced protection, antibodies may also provide prophylactic and therapeutic protection against pulmonary infection with low virulence *F. tularensis*, strains (Elkins et al., 2007; Metzger et al., 2007; Cole et al., 2009). Protective immunity induced by natural infection with *F. tularensis* or vaccination with LVS in humans depends primarily upon cell-mediated Th1-type immune responses, including interferon-gamma (IFN-γ), tumor necrosis factor-alpha (TNF-α), and interleukin-12 (IL-12) (Karttunen et al., 1987; Tarnvik, 1989; Sjostedt et al., 1992a; Ericsson et al., 1994, 2001).

## The LVS vaccine in human studies

The LVS vaccine was studied for safety and efficacy in small numbers of individuals in the 1960s. Saslaw et al. showed that individuals vaccinated with LVS intradermally (i.d., via multiple punctures) develop local lesions manifested by erythema (~1 cm in diameter) followed by non-tender papules that fade rapidly with no fever or systemic reactions (Saslaw et al., 1961a). Hornick et al. showed that among persons exposed to aerosolized LVS, 90% of individuals that inhaled 10^8^ LVS exhibited mild typhoidal tularemia and 80% had temperature elevations of >100°F; symptoms and signs were milder when the inhalation dose was reduced to 10^6^ (Hornick and Eigelsbach, 1966). Saslaw et al. further showed that i.d. vaccination of humans with LVS induced incomplete protection (3 of 18 persons developed disease vs. 8 of 10 nonvaccinated controls) against aerosol challenge with 10–53 CFU of subsp. *tularensis* SCHU S4 strain; in contrast, in a separate part of the study, the Foshay vaccine was not protective as there was no significant difference in the incidence of disease between the vaccinated and unvaccinated groups after aerosol challenge with similar doses of subsp. *tularensis* SCHU S4 strain (Saslaw et al., 1961a). Hornick and Eigelsbach demonstrated that individuals vaccinated with 10^6^ or 10^8^ CFU LVS by aerosol were well protected (none required antibiotic treatment) against a high dose challenge with aerosolized SCHU S4 (25,000 CFU, 500–2,500 minimum infective doses) 4–6 months post-vaccination. Similarly vaccinated volunteers were better protected than individuals immunized with a lower dose of LVS (10^4^ CFU) by aerosol or vaccinated i.d. by acupuncture; 46% of the latter group required antibiotic treatment vs. 89% of unvaccinated controls (Hornick and Eigelsbach, 1966). Hornick et al. also demonstrated that oral vaccination with LVS provided incomplete protection against aerosol challenge with SCHU S4 (Hornick et al., 1966). However, the i.d. route, but not the aerosol or oral route, has been regularly used for humans in the U.S.A. Saslaw and Carhart found that the greater efficacy of the live LVS vaccine than the Foshay killed vaccine was not related to antibody titers (Saslaw and Carhart, 1961). Recently, a new lot of LVS manufactured under modern GMP conditions has been studied in animals and in two human clinical trials to evaluate its safety, reactogenicity, and immunogenicity (Pasetti et al., 2008; El Sahly et al., 2009; Mulligan et al., 2017). In a Phase I study, Sahly et al. reported that individuals vaccinated with LVS by scarification or subcutaneously (s.c.) frequently experienced ≥grade 2 (interfering with activity) systemic complaints, especially headache and fatigue; many experienced erythema and induration at the injection site of which ~one-third had ≥grade 2 erythema (≥30 mm) and induration (≥15 mm). Of note, some individuals, especially those vaccinated by scarification, developed transient lymphangitis and papular satellite lesions surrounding the infection site. High dose scarification vaccination induced serologic immune responses and scarification and to a lesser extent s.c. vaccination induced high IFN-γ responses (El Sahly et al., 2009). In a Phase II study, Mulligan et al. compared the new lot of LVS to the existing USAMRIID LVS vaccine (Mulligan et al., 2017). They found that both vaccines appeared safe. Injection site reactogenicity was deemed generally mild and similar for the two vaccines; severe vaccine site reactions (erythema or induration >5 cm) occurred in 4.4 and 3.5% of vaccinees administered the new lot and USAMRIID vaccines, respectively. Similar percentages of the vaccinees admimistered the new lot or USAMRIID vaccine experienced severe (2 and 3%, respectively) or moderate (24 and 22%, respectively) systemic reactions. Both vaccines resulted in similar (94%) rates of seroconversion.

Thus, while LVS retains residual toxicity, it has demonstrated substantial albeit incomplete protection in humans against aerosol challenge with virulent subsp. *tularensis* SCHU S4. Although LVS has not been licensed for use in the U.S.A. and European Union, it is the only vaccine that has been shown to be reasonably safe and efficacious in humans. There is a general consensus that any vaccine that warrants further consideration as a human vaccine needs to be safer than LVS while providing protection comparable to or greater than that provided by LVS against aerosolized fully virulent *F. tularensis* subsp. *tularensis*. Because LVS retains significant virulence in animals, has toxicity in humans, and provides incomplete protection against aerosol challenge with SCHU S4, there has been great interest since 2001 in developing alternative vaccines—these are reviewed below and summarized in Tables 1–**5**. We focus here on vaccines that have been tested against subsp. *tularensis* SCHU S4 respiratory challenge and compared with LVS for efficacy and virulence.

**Table 1:** *F. tularensis* subsp. *holarctica* mutants: Protection against subsp. *tularensis* SCHU S4 respiratory challenge.

**Vaccine^a^**	**Host strain**	**Vaccine route, dose (CFU)^b^**	**Boost (route)**	**LVS control (route)**	**Interval (days)^c^**	**SCHU S4 challenge route, dose (CFU)**	**% Survival post-challenge (MST, days)^d^**			**References**
							**Vaccine**	**LVS**	**Sham**	
**NUTRIENT METABOLIC MUTANT**										
LVS Δ*purMCD*	BALB/c	i.n., 10^6^	No	Yes (i.n.)	42	i.n., 100	0 (6)	100 (21)	0 (5)	Pechous et al., 2008
						i.n., 2000	0 (5)	0 (7)	0 (5)	
	BALB/c	i.n., 10^6^	Yes (i.n.)	Yes (i.n.)	21	i.n., 100	100 (21)	100 (21)	0 (5)	Pechous et al., 2008
						i.n., 2000	33 (17)	33 (11)	0 (5)	
**OXIDATIVE STRESS RESPONSE MUTANTS**										
LVS Δ*sodB_*Ft*_*	C57BL/6	i.n., 5 × 10^3^	No	Yes (i.n.)	21	i.n., 14	40	0 (12)	0 (7)	Bakshi et al., 2006, 2008
	C57BL/6	i.n., 5 × 10^2^	Yes (i.n.)	Yes (i.n.)	21	i.n., 103	42	0 (15)	0 (6)	Bakshi et al., 2006, 2008
*emrA1*	C57BL/6	i.n., 10^6^	No	No	21	i.n., 32	0 (8)	ND	0 (6)	Suresh et al., 2015
		i.n., 10^6^	Yes (i.n.)	No	21	i.n., 38	0 (9)	ND	0 (6)	Suresh et al., 2015
		i.d., 10^6^	Yes (i.d.)	No	21	i.n., 17	0 (10)	ND	0 (7)	Suresh et al., 2015
		i.d., 10^6^	Yes (i.n.)	No	21	i.n., 17	0 (10)	ND	0 (7)	Suresh et al., 2015
		i.n., 10^3^	Yes (i.d.)	No	21	i.n., 23	20	ND	0 (6)	Suresh et al., 2015
		i.d., 10^3^	Yes (i.n.)	No	21	i.n., 24	20	ND	0 (6)	Suresh et al., 2015
Δ*dsbA*/FSC200	BALB/c	i.n., 10^2^	No	No	28	i.n., 100	0	ND	0 (5)	Straskova et al., 2015
		i.n. 10^3^	No	No	28	i.n., 100	0	ND	0 (5)	Straskova et al., 2015
		i.n., 10^4^	No	No	28	i.n., 100	20	ND	0 (5)	Straskova et al., 2015
		i.n., 10^5^	No	No	28	i.n., 100	30	ND	0 (5)	Straskova et al., 2015
		i.n., 10^6^	No	No	28	i.n., 100	50	ND	0 (5)	Straskova et al., 2015
**HEAT SHOCK PROTEIN MUTANTS**										
LVS Δ*clpB*	BALB/c	i.d., 10^5^	No	No	42	i.n. 40	0 (~12)	ND	0 (5)	Golovliov et al., 2013
FSC200 Δ*clpB*	BALB/c	i.d., 10^5^	No	Yes (i.d.)	42	i.n. 86	40	0	0 (5)	Golovliov et al., 2013
**CAPSULAR AND MEMBRANE MUTANTS**										
*LVS* Δ*capB*	BALB/c	i.d., 10^6^	No	Yes (i.d.)	42	Aero., 10	0 (10)	67 (16)	0 (5)	Jia et al., 2010
		i.n., 10^5^	No	Yes (i.n.)	42	Aero. 10	100 (21)	100 (21)	0 (5)	Jia et al., 2010
rLVS Δ*capB* /IglA	BALB/c	i.d., 10^6^	No	Yes (i.d.)	42	Aero., 10	50 (16)	63 (17)	0 (5)	Jia et al., 2013
rLVS Δ*capB* /IglC	BALB/c	i.d., 10^6^	No	Yes (i.d.)	42	Aero., 10	40 (14)	63 (17)	0 (5)	Jia et al., 2013
rLVS Δ*capB* /IglABC	BALB/c	i.d., 10^6^	No	Yes (i.d.)	42	i.n., 16–31	0	50	0 (4)	Jia et al., 2016
	BALB/c	i.d., 10^6^	Yes (i.d.)	Yes (i.d.)	42	i.n., 10	50	50-75	0 (4)	Jia et al., 2018
	BALB/c	i.n., 10^6^	Yes (i.n.)	Yes (i.d.)	42	i.n., 6-10	83-100	50-75	0 (4)	Jia et al., 2018
LVS::Δ*wzy*	BALB/c	i.n., 3.5 × 10^6^	Yes (i.n.)	Yes (i.n.)	28	i.n., 8	84	100	0 (6)	Kim et al., 2012
LVS::wbtA-OPS-TT^*^	BALB/c	i.n., 1.5 × 10^7^	Yes (i.n.)	No	28	i.n., 10	40 (15)	ND	20 (7)	Sebastian et al., 2009
LVS::Δ*wbtA*	BALB/c	i.n., 3.7 × 10^6^	Yes (i.n.)	Yes (i.n.)	28	i.n., 8	0	100	0 (6)	Kim et al., 2012
*FTL_0057*	BALB/c	i.n., 10^7^	No	No	30	i.n., 100	100	ND	0 (6)	Mahawar et al., 2013
*FTL_0325*	BALB/c	i.n., 10^7^	No	No	30	i.n., 100	100	ND	0 (6)	Mahawar et al., 2013
**OTHER MUTANTS**										
*LVS FTL0552* *(pmrA)*	BALB/c	i.n., 10^5^	No	No	30	i.n., 100	30	ND	0 (6)	Sammons-Jackson et al., 2008
	BALB/c	i.n., 10^5^	Yes (i.n.)	No	30	i.n., 100	40	ND	0 (6)	Sammons-Jackson et al., 2008
	C57BL/6	i.n., 10^4^	No	No	30	i.n., 100	0 (9)	ND	0 (7)	Sammons-Jackson et al., 2008
	C57BL/6	i.n., 10^4^	Yes (i.n.)	No	30	i.n., 100	0 (16)	ND	0 (7)	Sammons-Jackson et al., 2008
*FTL_0291*	BALB/c	i.n., 10^7^	No	No	30	i.n., 100	100	ND	0 (6)	Mahawar et al., 2013
*FTL_0304*	BALB/c	i.n., 10^7^	No	No	30	i.n., 100	0 (12)	ND	0 (6)	Mahawar et al., 2013

a Vaccine:

* *, animals vaccinated simultaneously with LVS::wbtA and OPS-TT [LVS O-polysaccharide (OPS) conjugated with–tetanus toxoid (TT)]*.

b *Vaccine route, dose: i.n., intranasal; i.d., intradermal*.

c *Interval: Interval between the only or the last vaccination and challenge*.

d *% Survival: % survival post-challenge in animals immunized with the vaccine candidate (vaccine), LVS control (LVS), or PBS or unvaccinated control (Sham); MST, Mean/Median Survival Time; ND, not determined*.

## Live attenuated subsp. *holarctica* vaccine candidates

Various mutant strains of subsp. *holarctica*, mainly in the background of LVS, have been developed, including mutants that have a lesion in a pathway or gene involved in nutrient metabolism (*purMCD*), response to oxidant stress (*sodB, emrA1*), the heat shock response (*clpB*), putative capsular synthesis (*capB*), membrane integrity (FTL_0325, FTL_0057, *wbtA, wzy*), transcription (*mglA)*, disulfide bond formation (*dsbA*), and other functions (Bakshi et al., 2006, 2008; Pechous et al., 2006, 2008; Meibom et al., 2008; Sebastian et al., 2009; Jia et al., 2010, 2013; Kim et al., 2012; Barrigan et al., 2013; Golovliov et al., 2013; Mahawar et al., 2013; Straskova et al., 2015; Suresh et al., 2015). Some of these strains have demonstrated significant attenuation by the intranasal (i.n.) route, and importantly, provided immune protection against respiratory challenge with virulent subsp. *tularensis* SCHU S4 strain (Table 1). The SCHU S4 strain of *F. tularensis* subsp. *tularensis* is used almost exclusively in vaccine testing. Of note, although the virulence of LVS in BALB/c mice is similar to that in C57BL mice, with an estimated LD_50_ in both strains of <10^3^ CFU by the i.n. route, < 10^1^ CFU by the intraperitoneal (i.p.) route, and >10^7^ CFU by the intradermal (i.d.) route (Saslaw et al., 1961a; Hornick and Eigelsbach, 1966; Fortier et al., 1991; Jia et al., 2009), the protective immunity induced by LVS is different in these two mouse strains (Chen et al., 2003). BALB/c mice immunized with LVS are protected against systemic challenge with both subsp. *tularensis* and subsp. *holarctica* strains; C57BL mice immunized with LVS are protected against systemic challenge with only the subsp. *holarctica* strain (Chen et al., 2003).

### Nutrient metabolic mutant

Pechous et al. constructed an LVS mutant with a deletion in the *purMCD* purine biosynthetic locus, LVS Δ*purMCD*, by allelic exchange (Pechous et al., 2006), and complemented the Δ*purMCD* strain *in trans* with wild type *purMCD*. The authors showed that the LVS Δ*purMCD* mutant is defective for growth in medium containing limiting concentrations of purines and is defective for intra-macrophage growth, as it escapes from the phagosome but fails to replicate in the cytosol. The Δ*purMCD* mutant is significantly attenuated in BALB/c mice with an LD_50_ >5 × 10^6^ by the i.p. route (vs. < 10^1^ CFU for LVS). 100% of BALB/c mice immunized i.p. with 5 × 10^4^-5 × 10^6^ CFU of the LVS Δ*purMCD* mutant and challenged 21 days later i.p. with parental LVS (5 × 10^1^-5 × 10^3^) survived the challenge, while 40% of naïve mice survived the challenge (Pechous et al., 2006). The authors furthered tested the efficacy of LVS Δ*purMCD*, with or without boosting, against respiratory challenge with SCHU S4 and compared it with LVS. In contrast to the result after challenge of the immunized mice with LVS i.p., mice immunized i.n. once with LVS Δ*purMCD* were not protected (0% survival) against i.n. challenge with SCHU S4, similar to sham-immunized mice, whereas mice immunized i.n. once with LVS had 100% survival. However, the protection was increased to the levels induced by LVS following a homologous booster vaccination (Table 1, *Nutrient metabolic mutant*) (Pechous et al., 2008). These studies underscore the fact that protection against challenge with subsp. *holartica* LVS is not predictive of protection against challenge with subsp. *tularensis* SCHU S4, especially when the vaccination and challenge routes are different, in this case i.p/i.p. vs. i.n./i.n.

### Oxidative stress response mutants

Several oxidant mutants of LVS have been reported (Lenco et al., 2005; Bakshi et al., 2006, 2008; Buchan et al., 2009; Melillo et al., 2009; Honn et al., 2012, 2017; Ma et al., 2014; Suresh et al., 2015; Saha et al., 2017) among which LVS mutants with a point mutation in *sodB* (FTL_1791), encoding an iron superoxide dismutase (*sodB*_Ft_), with a transposon insertion in a putative gene for the EmrA1 (FTL_0687) secretion protein (*emrA1*), or with a deletion in *dsbA*, encoding a disulfide oxidoreductases protein family homolog (ΔdsbA/FSC200), have been tested in mice for their efficacy against respiratory challenge with SCHU S4 (Bakshi et al., 2006, 2008; Straskova et al., 2015; Suresh et al., 2015). Both *sodB*_Ft_ and *emrA1* LVS mutants are sensitive to oxidant stress and are more attenuated than the parental LVS in mice; C57BL/6 and BALB/c mice infected i.n. with 1 × 10^4^ CFU of the *sodB*_Ft_ mutant of LVS had 83 and 60% survival, respectively, while C57BL/6 and BALB/c mice infected i.n. with 1 × 10^4^ CFU parental LVS had 8.3 and 0% survival, respectively (Bakshi et al., 2006). C57BL/6 mice immunized i.n. with 5 × 10^3^ s*odB*_Ft_ mutant and 21 days later challenged i.n. with 14 CFU SCHU S4 had 40% survival, significantly greater than that of naïve mice (0% survival) and mice immunized i.n. with 5 × 10^3^ LVS (0% survival). The protection was similar following administration of a homologous booster vaccination (42% survival) against i.n. challenge with 103 CFU of SCHU S4 (Table 1, *Oxidative stress response mutants*) (Bakshi et al., 2008). It is noted that the i.n. vaccination doses for the *sodB*_Ft_ mutant and LVS were both close to the LD_50_ i.n. and the interval between the last immunization and challenge was only 21 days.

The *emrA1* mutant of LVS is more attenuated than the *sodB*_Ft_ mutant of LVS in mice, with an LD_50_ > 10^6^ i.n. in C57BL/6 mice (Ma et al., 2014). However, mice immunized with 1 x10^6^ *emrA1* and challenged 21 days later i.n. with 32 CFU of the heterologous subsp. *tularensis* SCHU S4 strain had 0% survival; mice homologously primed-boosted with 1 × 10^6^ *emrA1* via the i.n. or the i.d. route or both routes alternately (i.d./i.n,. or i.n./i.d.) and challenged 21 days later had 0–20% survival, similar to naïve mice (Table 1, *Oxidative stress response mutants*). It is noted that mice immunized with *emrA1* had 100% survival after i.n. challenge with the homologous parental LVS strain at doses up to 1 × 10^8^ CFU LVS (Suresh et al., 2015). Thus, the vaccine was poorly protective against SCHU S4 but highly protective against LVS. These results suggest that over attenuation of LVS resulted in a significant reduction in protective immunity. Importantly, these results once again show that efficacy against respiratory challenge with subsp. *holartica* LVS does not predict efficacy against respiratory challenge with subsp. *tularensis* SCHU S4 (see Summary and Discussion).

The *dsbA* mutant of FSC200 (a fully virulent Type B strain genetically similar to LVS, with LD_100_ < 5 CFU i.d. and i.p. in mice), Δ*dsbA*/FSC200, administered i.n. has also shown dose-dependent protection against i.n. challenge with SCHU S4 (Table 1, *Oxidative stress response mutants*) (Straskova et al., 2015).

### Heat shock protein mutants

Several *F. tularensis* Type B (LVS and FSC200) mutants defective in heat shock chaperone protein ClpB, which is involved in the response to oxidative, ethanol, and acid stresses, have been reported (Meibom et al., 2008; Golovliov et al., 2013). LVS ClpB (FTL_0094) has 98–100% identity to ClpB in other *F. tularensis* strains (Meibom et al., 2008). The *clpB* mutant strains derived from subsp. *holarctica* LVS and FSC200 and subsp. *tularensis* SCHU S4 are attenuated when delivered by i.d., i.p., or oral routes; they have been tested as vaccine candidates against respiratory challenge with SCHU S4 (Meibom et al., 2008; Conlan et al., 2010; Shen et al., 2010; Twine et al., 2012; Golovliov et al., 2013; Ryden et al., 2013). Specifically, the LD_50_ for the LVS Δ*clpB* (transposon insertional mutant) is >10^7^ CFU i.p. in BALB/c mice (vs. < 10^1^ CFU for LVS) and the LD_50_ for FSC200 Δ*clpB* (deletional mutant) is >1 × 10^5^ CFU i.n. for BALB/c mice (vs. ~10^3^ CFU for LVS); however, FSC200 Δ*clpB* replicated to higher numbers at the intradermal vaccination site and was more lethal than LVS in SCID mice (Meibom et al., 2008; Golovliov et al., 2013). C57BL/6J (B6) mice immunized i.n. with 5 × 10^4^ LVS Δ*clpB* and challenged 28 or 120 days later with 5 × 10^3^ parental LVS i.n. (~5 LD_50_) had 100% survival, the same as mice immunized with 5 × 10^2^ LVS i.n.; all naïve mice died by day 7 (Barrigan et al., 2013). In a separate study, BALB/c mice immunized i.d. with 10^5^ CFU LVS Δ*clpB* and challenged 6 weeks later i.n. with 40 CFU SCHU S4 had 0% survival (Golovliov et al., 2013). BALB/c mice immunized i.d. with 10^5^ CFU FSC200 Δ*clpB* and challenged 6 weeks later i.n. with 86 CFU SCHU S4 had ~40% survival vs. 0% for mice immunized i.d. with LVS (Golovliov et al., 2013). Both FSC200 Δ*clpB* and LVS Δ*clpB* mutants were generally less protective than a SCHU S4 Δ*clpB* mutant against i.n. challenge with SCHU S4 (Table 1, **Table 3**, *Heat shock protein mutants*), although the latter strain has only a single deletional mutation and thus presents safety issues (see Summary and Discussion).

### Putative capsular and membrane mutants

*F. tularensis* Type B mutants defective in genes involved in putative capsular and membrane synthesis are highly attenuated in mice, including LVS mutants defective in *capBCA* (FTT0806, FTT0805, and FTT0798, respectively), *wzy, wbtA*, FTL_0057, and FTL_0325 (Table 1, Capsular and membrane mutants) (Sebastian et al., 2007; Su et al., 2007, 2011; Weiss et al., 2007; Jia et al., 2010; Michell et al., 2010; Kim et al., 2012; Mahawar et al., 2013). We have constructed and characterized a defined LVS mutant with an antibiotic resistance marker-free deletion in *capB (FTL_1416)* (LVS Δ*capB*) as a vaccine candidate and shown that LVS Δ*capB* is resistant to serum killing, out-competed for growth by its parental LVS in infected human macrophage cells, and significantly more attenuated (>10,000-fold) than LVS administered i.n. in mice (Jia et al., 2010). BALB/c mice immunized with LVS Δ*capB* i.n. or i.d. develop humoral and cellular immune responses comparable to LVS and induce full protection (100% survival) against i.n. challenge 4 or 8 weeks later with the parental LVS vaccine (~5 LD_50_). Most importantly, while the vaccine was poorly protective when administered i.d., mice immunized with LVS Δ*capB* i.n. were highly protected (100% survival) against an aerosol challenge 6 weeks later with 10 × LD_50_ *F. tularensis* SCHU S4 (Table 1, *Capsular and membrane mutant*) (Jia et al., 2010). In subsequent studies, we constructed several rLVS Δ*capB* strains over-expressing T6SS proteins IglA (rLVS Δ*capB/iglA)* or IglC (rLVS Δ*capB/iglC)* or a fusion protein comprising immunoprotective domains of IglA, IglB, and IglC (rLVS Δ*capB/iglABC*) and evaluated their capacity to protect against i.n. challenge (more consistent route than aerosol challenge) with *F. tularensis* SCHU S4. BALB/c mice immunized once i.d. with rLVS Δ*capB*/iglC or rLVS Δ*capB*/iglA had a significantly greater survival rate (40 and 50%, respectively) than mice immunized with the parental LVS Δ*capB* vector (0% survival, *p* = 0.09 and 0.01, respectively), which was not significantly different from that of the LVS-immunized mice (63% survival) (Table 1, *Capsular and membrane mutant*) (Jia et al., 2013). In separate studies, mice homologously primed-boosted with rLVS Δ*capB*/*iglABC* i.n. three times at Weeks 0, 4, and 8 or twice at Weeks 4 and 8 and challenged i.n. at Week 14 with a lethal dose of SCHU S4 had 83–100% survival—greater than LVS i.d. vaccination—while all the naïve mice died at day 4 post-challenge. Mice homologously primed-boosted i.d. with rLVS Δ*capB*/*iglABC* twice or three times had survival equivalent to LVS-immunized mice (Table 1, *Capsular and membrane mutants*) (Jia et al., 2016, 2018).

Kim et al. constructed *wzy* (O-antigen polymerase) (LVS::Δ*wzy*) and *wbtA* (LVS::Δ*wbtA*) deletional mutants of LVS; both were significantly attenuated in mice (Kim et al., 2012). BALB/c mice immunized i.n. with LVS::Δ*wzy*, but not with LVS::Δ*wbtA*, were highly protected against i.n. challenge 4 weeks later with 8 CFU SCHU S4, similar to LVS i.n. immunized mice (Table 1, *Capsular and membrane mutants*) (Kim et al., 2012). Mahawa et al. developed LVS mutants defective in a conserved hypothetical membrane protein (FTL_0057) or in an outer membrane protein A-like family protein (FTL_0325). BALB/c mice immunized i.n. with FTL_0057 or FTL_0325 mutants of LVS were highly protected against i.n. challenge with SCHU S4; however, their efficacy relevant to LVS was not studied (Table 1, *Capsular and membrane mutants*) (Mahawar et al., 2013).

### Other subsp. *holarctica* mutants

Subsp. *holarctica* LVS mutants deficient in FTL_0552 (a transcriptional response regulation gene, *pmrA*), FTL_0291, or FTL_0304 also have shown attenuated phenotypes and protective efficacy against i.n. challenge with SCHU S4 in murine models. However, these mutants were not compared for efficacy with the parental LVS vaccine (Table 1, *Other mutants*) (Sammons-Jackson et al., 2008; Mahawar et al., 2013).

## Live attenuated *F. novicida* vaccine candidates

*F. novicida* vaccine candidates have been constructed by deleting genes involved in the purine biosynthesis pathway (*purA* and *purF*), the T6SS (*iglB, iglC, pdpB*, and *iglD*), the response regulator (*pmrA*), or in other activities. A complete list of the *F. novicida* mutants can be found in a review article written by Pechous et al. (2009). However, only a few of the *F*. *novicida* mutants, including mutants with a deletion in *purF, purA, iglB, iglD*, or *pmrA*, have been tested against challenge with virulent SCHU S4, and even fewer against respiratory challenge with SCHU S4. In one study, BALB/c mice immunized i.p., but not s.c., with *F. novicida* U112Δ*purF*::cm were partially protected against i.p. challenge with the homologous U112 strain, but not against challenge with the heterologous SCHU S4 strain; a similar mutant, U112Δ*purA*::cm, was not protective against either U112 or SCHU S4 challenge (Quarry et al., 2007). Two *F. novicida* U112 *iglB* deletional mutants, U112Δ*iglB* and U112Δ*iglB*::fljB (expressing one domain D of the *Salmonella typhimurium* FljB flagellin, reportedly a TLR5 agonist), administered orally, have been shown to partially protect against i.n. or intratracheal (i.t.) challenge with SCHU S4 in mice and Fisher rats; however, efficacy was not compared with LVS (Table 2; Cong et al., 2009; Signarovitz et al., 2012; Cunningham et al., 2014). An *F. novicida* mutant with a deletion in *iglD* (Fn IglD), although not protective in a murine model of tularemia, has demonstrated protection against i.t. challenge with SCHU S4 in the more resistant Fisher rat model and in the non-human primate model of tularemia (see below for details) (Chu et al., 2014). Another *F. novicida* mutant with a deletion in an orphan response regulator gene *pmfA* was protective against homologous U112 but not against heterologous SCHU S4 challenge (Table 2; Mohapatra et al., 2007). Other studies also have shown that *F. novicida* and its derivative mutants can induce protective immunity against homologous respiratory challenge with wild-type *F. novicida*; however, these mutants have not been shown capable of inducing protection against respiratory challenge with subsp. *tularensis* in murine models of pneumonic tularemia by traditional i.d. or i.n. routes (Pammit et al., 2006; Sanapala et al., 2012).

**Table 2:** *F. novicida* mutants: Protection against *F. tularensis* subsp. *tularensis* challenge.

**Vaccine**	**Host strain^a^**	**Vaccine route, dose (CFU)^b^**	**Boost (route)**	**LVS control (route)**	**Interval (days)^c^**	**SCHU S4 challenge route, dose (CFU or LD_50_)^d^**	**% Survival post-challenge (MST, days)^e^**			**References**
							**Vaccine**	**LVS**	**Sham**	
U112	BALB/c	i.d., 100	No	Yes (i.d.)	56	Aerosol, 10^§^	0 (5)	0 (6)	0 (5)	Shen et al., 2004
U112	BALB/c	i.d., 100	No	Yes (i.d.)	56	i.d., 350^§^	0 (5)	100 (>21)	0 (6)	
U112	Rats	i.t., 10^7^	No	No	30	i.t., 25 LD_50_	100	ND	0 (10)	Signarovitz et al., 2012
		p.o., 10^7^	No	No	30	i.t., 25 LD_50_	50	ND	0 (10)	Signarovitz et al., 2012
Δ*iglB* (KKF235)	C57BL/6	p.o., 10^3^	No	No	21	i.n., 25	67	ND	0 (6)	Cong et al., 2009
	C57BL/6	p.o., 10^3^	No	No	21	i.n., 50	10	ND		Cong et al., 2009
	C57BL/6	p.o., 10^3^	Yes (p.o.)	No	21	i.n., 52	40	ND		Cong et al., 2009
Δ*iglB*	Rats	i.t., 10^7^	No	No	30	i.t., 1.25 × 10^4*^	50	ND	0 (10)	Signarovitz et al., 2012
		p.o., 10^7^	No	No	30	i.t., 1.25 × 10^4*^	50	ND	0 (10)	Signarovitz et al., 2012
Δ*iglB*::fljB	BALB/c	p.o., 10^7^	No	No	30	i.t., 1.25 × 10^4*^	83	ND		Cunningham et al., 2014
	Rats	p.o., 10^7^	No	No	30	i.t., 1.25 × 10^4*^	88	ND		Cunningham et al., 2014
Fn *iglD*	BALB/c	i.n., ~10^9^	No	No	30	i.n., 10^3^	0	ND	0	Chu et al., 2014
	Rats	p.o., 10^7^	No	No	30	i.t., 10^4^	83	ND	17	Chu et al., 2014
	Rats	i.t., 10^5^	No	No	30	i.t., 10^4^	100	ND	25	Chu et al., 2014
	Rats	i.t., 10^7^	No	No	30	i.t., 10^4^	83	ND	25	Chu et al., 2014
	NHP	t.b., 10^8^	No	Yes (s.c.)	30	Aerosol, (2,500–5,000)	83	100	0 (8)	Chu et al., 2014
Δ*pmrA*	BALB/c	i.n. (10^6^)	No	No	35	i.n., 100	Not protected	ND	ND	Mohapatra et al., 2007

a *Host strain: Mice: BALB/c or C57BL/6 mice; Rats: Fisher rats; NHP: non-human primates, cynomolgus macaques*.

b *Vaccine route: intranasal (i.n.); oral (p.o.); intratracheal (i.t.); via bronchoscopy (t.b.)*.

c *Interval: interval between the only or the last vaccination and challenge*.

d SCHU S4 challenge route (dose, CFU of LD50):

§ challenged with subsp. tularensis FSC 033 strain; *,1.25 × 10^4^ CFU, approximately 25 LD_50_ in rats.

e *% Survival post-challenge: % survival post-challenge in animals immunized with the vaccine candidate (vaccine), LVS control (LVS), or PBS or unvaccinated control (Sham). MST, mean/median survival time. ND; not determined*.

### T6SS mutant

The Francisella pathogenicity island (FPI) proteins, including IglD, are required for bacterial phagosome escape, intracellular replication, and virulence, and are components of a T6SS apparatus (Clemens et al., 2015). Chu et al. reported on the protective immunity induced by *iglD* deletion mutants of subsp. *tularensis* (Ftt *iglD*) and *F. novicida* (Fn *iglD*) in murine, Fischer rat, and non-human primate models of pneumonic tularemia (Chu et al., 2014). Ftt *iglD* and Fn *iglD* are defective for intramacrophage replication and attenuated in mice (the LD_50_ for Fn iglD is 9.7 × 10^8^ CFU i.n. in BALB/c mice vs. ~10^3^ CFU for LVS). Mice immunized i.n. with Fn iglD were fully protected against subsequent homologous challenge with 10^3^ CFU of the parental *F. novicida* U112 strain; however, mice immunized i.n. with Fn *iglD* or Ftt *iglD* were not protected against respiratory challenge with the heterologous subsp. *tularensis* SCHU S4 strain. Arguing that the mouse may be too sensitive to tularemia, Chu et al. evaluated the Fn iglD vaccine in the Fischer rat, an animal model that has been shown to be more resistant to various *Francisella* subspecies than mice and non-human primates. Fischer rats vaccinated with Fn iglD orally or i.t. and challenged i.t. 30 days later with subsp. *tularensis* SCHU S4 had 83–100% survival post-challenge vs. 17–25% survival post-challenge for naïve rats. Of note, Fn *iglD* was not compared with LVS for efficacy in the murine and rat models. Cynomolgus macaques vaccinated with 10^8^ CFU Fn *iglD* via bronchoscopy and challenged via head-only aerosol inhalation 30 days later with ~2,500–5,000 CFU SCHU S4 had 83% survival, somewhat less than those immunized s.c with 10^8^ CFU LVS (100% survival); mock vaccinated animals died 7–13 days post-challenge (Table 2; Chu et al., 2014).

## Live attenuated subsp. *tularensis* vaccine candidates

Because subsp. *tularensis* and subsp. *holarctica* differ in genetic organization, antigen expression, and disease pathogenesis, it was hypothesized that attenuated mutants on the background of *subsp. tularensis* may offer better protection against respiratory challenge with the parental subsp. *tularensis* than mutants derived from subsp. *holarctica* (Conlan et al., 2003; Wu et al., 2005). Thus, a series of mutants have been generated in the SCHU S4 background with mutations previously shown to attenuate LVS and tested in murine, rabbit, and non-human primate models of pneumonic tularemia (Twine et al., 2005, 2012; Pechous et al., 2008; Qin et al., 2008, 2009; Conlan et al., 2010; Michell et al., 2010; Shen et al., 2010; Rockx-Brouwer et al., 2012; Reed et al., 2014; Santiago et al., 2015). Summarized below are some SCHU S4 mutants that have been tested against respiratory SCHU S4 challenge.

### Nutrient metabolic mutants

Based on the capacity of the attenuated LVS Δ*purMCD* mutant to induce protective immunity, Pechous et al. generated a defined subsp. *tularensis* SCHU S4 Δ*purMCD* mutant and showed that it is significantly more attenuated in mice when delivered by the i.d. (LD_50_ > 10^6^ CFU) and i.n. (LD_50_ > 10^6^ CFU) routes than its parental SCHU S4 strain (LD_50_ < 10 CFU i.n. or i.d.) (Pechous et al., 2008). However, mice immunized i.n. with SCHU S4 Δ*purMCD* had tissue damage in the lung and were protected no better than mice vaccinated with LVS or with an analogous LVS Δ*purMCD* mutant. BALB/c mice immunized once i.n. with 10^4^ CFU SCHU S4 Δ*purMCD*, 10^6^ CFU LVS Δ*purMCD* or 10^2^ CFU LVS and challenged 42 days later i.n. with 100 CFU SCHU S4 had 14, 0, and 100% survival, respectively; mice immunized twice i.n. with the same vaccines had 71, 100, and 100% survival after i.n. challenge with 100 CFU SCHU S4 (Results for SCHU S4 mutant and LVS shown in Table 3, *Nutrient metabolic mutants*) (Pechous et al., 2008); thus homologous i.n. boosting substantially improved the efficacy of the SCHU S4 Δ*purMCD* vaccine. Several other SCHU S4 mutants that are deficient in nutrient metabolic enzymes have also been tested against respiratory challenge with SCHU S4 in a murine or rat model (Table 3, *Nutrient metabolic mutants*) (Reed et al., 2014; Santiago et al., 2015).

**Table 3:** *F. tularensis* subsp. *tularensis* SCHU S4 mutants: Protection against SCHU S4 respiratory challenge.

**Vaccine**	**Host strain^a^**	**Vaccine route, dose (CFU)**	**Boost (route)**	**LVS control (route)**	**Interval (days)^b^**	**SCHU S4 challenge route, dose (CFU or LD_50_)^c^**	**% Survival post-challenge (MST, days)^d^**			**References**
							**Vaccine**	**LVS**	**Sham**	
**NUTRIENT METABOLIC MUTANTS**										
Δ*purMCD*	BALB/c	i.n., 10^4^	No	Yes (i.n.)	42	i.n., 100	14 (11)	100 (21)	0 (5)	Pechous et al., 2008
		i.n., 10^4^	No	Yes (i.n.)	42	i.n., 2000	0 (6)	0 (7)	0 (5)	Pechous et al., 2008
		i.n., 10^4^	Yes (i.n.)	Yes (i.n./i.n.)	21	i.n., 100	71 (18)	100 (21)	0 (5)	Pechous et al., 2008
		i.n., 10^4^	Yes (i.n.)	Yes (i.n./i/n.)	21	i.n., 2000	0 (7)	33 (11)	0 (5)	Pechous et al., 2008
		i.d., 10^1−6^	No	No	21	i.n., 500	0 (6-8)	ND	0 (6)	Pechous et al., 2008
		i.n., 10^1−6^	No	No	21	i.n., 500	0 (6-11)	ND	0 (6)	Pechous et al., 2008
ΔFTT1019c (*guaA)*	C57BL/6	i.n., 7 × 10^5^	No	No	28	i.n., 100	0 (4)	ND	0 (4)	Santiago et al., 2015
ΔFTT1317c (*guaB)*	C57BL/6	i.n., 1 × 10^9^	No	No	28	i.n., 95	0 (6)	ND	0 (4)	Santiago et al., 2015
		i.n., 6 × 10^7^	Yes (i.n.)	No	28	i.n., 100	0 (4)	ND	0 (4)	Santiago et al., 2015
ΔFTT1019c, ΔFTT1317c (*guaA, guaB*)	C57BL/6	i.n., 1 × 10^8^	Yes (i.n.)	No	28	i.n., 100	0 (4)	ND	0 (4)	Santiago et al., 2015
Δ*guaBA*	Rabbits	i.d., 10^9^	No	Yes (i.d.)	30	Aero., 40 LD_50_	27 (7)^*^	0 (7)	0 (5)	Reed et al., 2014
Δ*aroD*	Rabbits	i.d., 10^9^	No	Yes (i.d.)	30	Aero., 40 LD_50_	36 (7)^*^	0 (7)	0 (5)	Reed et al., 2014
**HEAT SHOCK PROTEIN MUTANTS**										
Δ*clpB*	BALB/c	i.d., 10^5^	No	Yes (i.d.)	42	Aero., 20	60 (28)	0 (8)	0 (5)	Conlan et al., 2010
	BALB/c	i.d., 10^5^	Yes (p.o.)	Yes (i.d./p.o)	42	Aero., 20	20 (11)	0 (8)	0 (5)	Conlan et al., 2010
	C3H/HeN	i.d., 10^5^	No	Yes (i.d.)	42	Aero., 20	0 (10)	0 (11)	0 (5)	Conlan et al., 2010
	C3H/HeN	i.d., 10^5^	Yes (p.o.)	Yes (i.d./p.o)	42	Aero., 20	0 (16)	0 (7)	0 (6)	Conlan et al., 2010
	BALB/c	p.o., 10^8^	No	Yes (p.o.)	42	Aero., 20	40 (16)	0 (5)	0 (5)	Conlan et al., 2010
	BALB/c	p.o., 10^8^	Yes (p.o.)	Yes (p.o./p.o)	42	Aero., 20	20 (11)	0 (7)	0 (5)	Conlan et al., 2010
	C3H/HeN	p.o., 10^8^	No	Yes (p.o.)	42	Aero., 20	0 (12)	0 (5)	0 (5)	Conlan et al., 2010
	C3H/HeN	p.o., 10^8^	Yes (p.o.)	Yes (p.o./p.o)	42	Aero., 20	60 (28)	0 (7)	0 (5)	Conlan et al., 2010
	BALB/c	i.d., 10^5^	No	Yes (i.d.)	42	i.n., 10	100	30	0 (~5)	Shen et al., 2010
	BALB/c	i.d., 10^5^	No	Yes (i.d.)	42	i.n., 100	80	0	0 (~5)	Shen et al., 2010
	BALB/c	i.d., 10^5^	No	Yes (i.d.)	42	i.n., 1000	20	0	0 (~5)	Shen et al., 2010
	BALB/c	i.d., 10^5^	No	No	42	i.n., 100	~75	ND	0 (~5)	Twine et al., 2012
	C57BL/6	i.d., 10^5^	No	No	42	i.n., 100	0	ND	0 (5)	Twine et al., 2012
	BALB/c	i.d., 10^3^	No	No	42	i.n., 105	~100	ND	0 (5)	Golovliov et al., 2013
	BALB/c	i.d., 10^5^	No	No	42	i.n., 105	~80	ND	0 (5)	Golovliov et al., 2013
	BALB/c	i.d., 10^7^	No	No	42	i.n., 105	~60	ND	0 (5)	Golovliov et al., 2013
	BALB/c	i.d., 10^5^	No	No	42	i.n., 40	100	ND	0 (~5)	Golovliov et al., 2013
Δ*clpB*Δ*capB*	BALB/c	i.d., 10^7^	No	Yes (i.d.)	42	Aero., 100	≤ 20	0	ND	Golovliov et al., 2013
**CAPSULAR MUTANT**										
ΔFTT0918 Δ*capB*	BALB/c	i.d., 10^3^	No	Yes (i.d.)	42	Aero., 2	40 (8)	0 (8)	0 (5)	Conlan et al., 2010
		i.d., 10^3^	Yes (p.o.)	Yes (i.d./p.o)	42	Aero., 2	0 (8)	0 (8)	0 (5)	Conlan et al., 2010
		p.o., 10^8^	No	Yes (p.o.)	42	Aero., 2	0 (5)	0 (5)	0 (5)	Conlan et al., 2010
		p.o., 10^8^	Yes (p.o.)	Yes (i.d./p.o.)	42	Aero., 2	0 (8)	0 (7)	0 (5)	Conlan et al., 2010
	C3H/HeN	i.d., 10^3^	No	Yes (i.d.)	42	Aero., 20	0 (5)	0 (11)	0 (5)	Conlan et al., 2010
		i.d., 10^5^	Yes (p.o.)	Yes (i.d./p.o.)	42	Aero., 20	50 (17)	0 (7)	0 (6)	Conlan et al., 2010
		p.o., 10^8^	No	Yes (p.o.)	42	Aero., 20	0 (5)	0 (5)	0 (5)	Conlan et al., 2010
		p.o., 10^8^	Yes (p.o.)	Yes (p.o./p.o.)	42	Aero., 20	0 (5)	0 (7)	0 (6)	Conlan et al., 2010
**LIPOPROTEIN MUTANTS**										
ΔFTT1103	C57BL/6	i.n., 3 × 10^7^	No	No	32	i.n., 68	100	ND	0 (5)	Qin et al., 2009
		i.n., 1 × 10^8^	No	No	32	i.n., 37	100	ND	0 (5)	Qin et al., 2009
		i.n., 3 × 10^8^	No	No	32	i.n., 68	50 (18)	ND	0 (5)	Qin et al., 2009
	BALB/c	i.n., 1 × 10^8^	No	No	33	i.n., 95	75 (21)	ND	0 (5)	Qin et al., 2009
ΔFTT1103 (*fipB*)	Rabbits	i.d., 10^9^	No	Yes (i.d.)	30	Aero., 40 LD_50_	0 (6)	0 (7)	0 (5)	Reed et al., 2014
**OTHER MUTANTS**										
ΔFTT0918	BALB/c	i.d., 10^5^	No	Yes (i.d.)	63	Aero., 10^*^	33 (15)	0 (7)	0 (5)	Twine et al., 2005
Δ*iglC*	BALB/c	i.d., 10^7^	No	Yes (i.d.)	63	Aero., 10^*^	0 (6)	0 (7)	0 (5)	Twine et al., 2005
Δ*iglC*	BALB/c	i.d., 10^5^	No	Yes (i.d.)	42	Aero., 20	0 (6)	0 (8)	0 (5)	Conlan et al., 2010
	BALB/c	p.o., 10^8^	No	Yes (p.o.)	42	Aero., 20	0 (5)	0 (5)	0 (5)	Conlan et al., 2010
	C3H/HeN	i.d., 10^5^	No	Yes (i.d.)	42	Aero., 20	0 (5)	0 (11)	0 (5)	Conlan et al., 2010
	C3H/HeN	p.o., 10^8^	No	Yes (p.o.)	42	Aero., 20	0 (5)	0 (5)	0 (5)	Conlan et al., 2010
ΔFTT0369c	BALB/c	i.d., 50	No	No	45	i.n., 10	90	ND	0 (5)	Rockx-Brouwer et al., 2012
		i.n., 10	No	No	45	i.n., 10	80	ND	0 (5)	Rockx-Brouwer et al., 2012
ΔFTT1676	BALB/c	i.d., 50	No	No	45	i.n., 10	100	ND	0 (5)	Rockx-Brouwer et al., 2012
		i.n., 10	No	No	45	i.n., 10	~50	ND	0 (5)	Rockx-Brouwer et al., 2012
ΔFTT0369 ΔFTT1676	BALB/c	i.d., 50	No	No	45	i.n., 10	60	ND	0 (5)	Rockx-Brouwer et al., 2012
		i.n., 10	No	No	45	i.n., 10	10	ND	0 (5)	Rockx-Brouwer et al., 2012

a *Host strain: Mice: BALB/c, C57BL/6, or C3H/HeN mice; Rabbits: New Zealand White rabbits*.

b *Interval: time between the only or the last vaccination and challenge*.

c SCHU S4 challenge route: ^*^challenged with subsp. tularensis (Type A) FSC033 strain.

d Survival: % survival after challenge of mice immunized with the vaccine candidate (vaccine), LVS control (LVS), or no vaccine or PBS control (Sham). MST, mean/median survival time;

* *Time to death does not include survivors; ND, not determined*.

### Heat shock mutants

A defined SCHU S4 *clpB* mutant (SCHU S4Δ*clpB*) has been studied extensively for its capacity to induce protective immunity in BALB/c, C3H/HeN, and C57BL/6 mice (Conlan et al., 2010; Shen et al., 2010; Twine et al., 2012; Golovliov et al., 2013). Conlan et al. immunized BALB/c mice or C3H/HeN mice with 1 × 10^5^ CFU i.d. or 1 × 10^8^ CFU orally (p.o.) with LVS or SCHU S4Δ*clpB*, boosted or did not boost p.o., and challenged the mice 6 weeks later by aerosol with 20 CFU of the parental SCHU S4 strain. Unvaccinated mice served as controls. BALB/c mice immunized i.d. or orally with SCHU S4Δ*clpB* had 60 and 40% survival, respectively, significantly greater than those of naïve mice (0% survival) and mice immunized with LVS (0% survival), whereas C3H/HeN mice immunized i.d. or orally with the same vaccine had 0% survival. Boosting i.d. immunized BALB/c and C3H/HeN mice p.o. at week 8 did not improve the survival of mice against SCHU S4 challenge; boosting orally immunized C3H/HeN, but not BALB/c mice, with SCHU S4Δ*clpB* did improve protection against SCHU S4 challenge (Conlan et al., 2010). Others also showed similar protective immunity of SCHU S4Δ*clpB* in BALB/C but not in C57BL/6 mice (Shen et al., 2010; Twine et al., 2012; Golovliov et al., 2013). Introducing a second deletional mutation—one in *capB*—to SCHU S4Δ*clpB (*SCHU S4Δ*clpB*Δ*capB*) appeared to reduce its capacity to induce protective immunity against respiratory challenge with parental SCHU S4 (Golovliov et al., 2013; Table 3, *Heat shock protein mutants*).

### Putative capsular mutants

Defined subsp. *tularensis* SCHU S4 mutants with a single deletion in *capB* (Δ*capB*::Cam) or double deletions in *FTT0918* and *capB* (Δ*FTT0918*Δ*capB)* have been developed by Michell and Conlan et al. and evaluated as vaccine candidates (Conlan et al., 2010; Michell et al., 2010). Michell et al. showed that the s.c. median lethal dose for SCHU S4 Δ*capB*::Cam, a single *capB* deletion mutant with a chloramphenicol resistance cassette (Cam) inserted at the deleted *capB* locus, is >1.6 × 10^6^ CFU in BALB/c mice. The level of protection afforded by s.c. immunization with 10^4^ SCHU S4Δ*capB*::Cam is comparable to that of s.c. immunization with 10^4^ CFU LVS against systemic (s.c.) challenge with 10^3^ SCHU S4 56 days later. The SCHU S4Δ*capB*::Cam vaccine was not tested against respiratory challenge (Michell et al., 2010). Conlan et al. showed that SCHU S4 Δ*FTT0918*Δ*capB* administered i.d. is as attenuated as LVS in BALB/c mice (LD_50_ ≥ 10^7^ CFU) but more virulent than LVS in C3H/HeN mice (LD_50_ < 10^5^ CFU vs. LD_50_ > 10^5^ CFU for LVS). In a virulence study, BALB/c and C3H/HeN mice infected orally with 10^8^ CFU SCHU S4 Δ*FTT0918*Δ*capB* had 80% (12/15) and 60% (9/15) survival, respectively (Conlan et al., 2010). In a study summarized in Table 3, *Capsular mutant*, BALB/c or C3H/HeN mice were not immunized, or immunized i.d. with 10^3^ CFU or orally with 10^8^ CFU Δ*FTT0918*Δ*capB* or LVS, homologously boosted or not boosted orally, and challenged by aerosol 6 weeks later with 20 CFU SCHU S4. BALB/c mice immunized i.d. or orally with Δ*FTT0918*Δ*capB* had 40 and 0% survival, respectively, after SCHU S4 aerosol challenge whereas none of the C3H/HeN mice immunized i.d. or orally with Δ*FTT0918*Δ*capB* survived the challenge; nor did the naïve mice or mice immunized i.d. or orally with LVS. Homologous boosting orally did not significantly improve the protection against aerosol challenge with SCHU S4 except that C3H/HeN mice immunized i.d. and boosted orally with Δ*FTT0918*Δ*capB* had improved survival (50% vs. 0% without boosting) (Conlan et al., 2010). In an earlier study, Twine et al. showed that the SCHU S4 FTT0918 single deletional mutant (SCHU S4 ΔFTT0918) had an i.d. LD_50_ of ~10^5^ CFU and was thus 10-fold more virulent than LVS in BALB/c mice. BALB/c mice immunized i.d. with 10^5^ Δ*FTT0918* and challenged i.n. 9 weeks later with 10 CFU FSC 033 had 33% survival, while LVS-immunized mice had 0% survival after aerosol FSC033 challenge (Table 3, *Other mutants*) (Twine et al., 2005). The results of these studies suggest that single deletional mutants in the background of SCHU S4, although more attenuated than the parental SCHU S4 strain, are generally still more virulent than LVS. Introducing a second major attenuating deletion attenuates the mutant further but diminishes its capacity to induce protective immunity such that it is comparable to or less protective than LVS vaccination against virulent subsp. *tularensis* challenge.

### Lipoprotein mutants

Qin et al. initially reported a subsp. *tularensis* SCHU S4 mutant with a transposon insertion at the FTT1103 locus (encoding a hypothetical lipoprotein); subsequently, they generated a defined subsp. *tularensis* SCHU S4 FTT1103 deletion mutant (SCHU S4 ΔFTT1103) (Qin et al., 2009, 2011). SCHU S4 ΔFTT1103 is highly attenuated in mice with an LD_50_ > 10^10^ i.n., and > 10^6^ i.p., s.c., or i.v. C57BL/6 mice immunized once i.n. with 3 × 10^7^, 1 × 10^8^, or 3 × 10^8^ SCHU S4 ΔFTT1103 and challenged i.n. 32 days later with 37–68 CFU SCHU S4 had 100%, 100% and 50% survival post-challenge; BALB/c mice immunized i.n. with 1 × 10^8^ SCHU S4 ΔFTT1103 and challenged i.n. 33 days later with 95 CFU of the parental SCHU S4 strain had 75% survival post-challenge; however, there was no LVS control included in this study (Table 3, *Lipoprotein mutants*) (Qin et al., 2009, 2011). Recently, Reed et al. evaluated the vaccine efficacy of SCHU S4 ΔFTT1103 along with several other SCHU S4 mutants in a rabbit model (Reed et al., 2014). New Zealand White (NZW) rabbits were vaccinated via scarification with PBS, LVS, SCHU S4 Δ*fibB* (FTT1103), SCHU S4 Δ*guaBA*, or SCHU S4 Δ*aroD*; 30 days later, the animals were challenged with 1,000–10,000 CFU (~40–400 LD_50_) of aerosolized SCHU S4 and monitored for signs of illness and survival. Animals vaccinated with SCHU S4 Δ*guaBA* or SCHU S4 Δ*aroD* had 27 and 36% survival, respectively (Table 3, *Nutrient metabolic mutants*); none of the mock-, LVS-, and Δ*fibB*-vaccinated animals survived the SCHU S4 challenge (Table 3, *Lipoprotein mutants*) (Reed et al., 2014).

### Other subsp. *tularensis* mutants

Other subsp. *tularensis* mutants, including SCHU S4 mutants with a single deletion in FTT0918, *iglC*, FTT0369, or FTT1676, or double deletions in FTT0369 and FTT1676 have also been reported; some have shown protection against i.n. challenge with SCHU S4 (Table 3, *Other mutants*) (Twine et al., 2005; Conlan et al., 2010; Rockx-Brouwer et al., 2012).

## Other live attenuated recombinant vaccine candidates

Other live attenuated recombinant vaccine candidates have been developed by using heterologous vectors—adenovirus, Tobacco Mosaic Virus, and *Listeria monocytogenes* (Lm), or a homologous LVS mutant (LVS Δ*capB* or LVS ΔLPS) vector to express/overexpress *F. tularensis* antigens (Jia et al., 2009, 2013, 2016; Kaur et al., 2012; Banik et al., 2015). Among these vaccines, some of the Lm-, LVS Δ*capB-*, and LVS ΔLPS*-*vectored vaccines were tested against respiratory challenge with subsp. *tularensis* SCHU S4 and these are summarized in Table 4. We constructed seven recombinant Lm (rLm) vaccines using Lm Δ*actA* (a live attenuated *actA* deficient mutant) as a vector to express *F. tularensis* proteins, including T6SS protein IglC, metabolic enzymes AcpA, KatG, and Pld, and other proteins including bacterioferritin (Bfr), DnaK, and GroEL, and tested their protective immunity against LVS i.n. challenge in BALB/c mice. Among the seven rLm vaccines constructed, two of them, rLm/iglC and rLm/katG, were further tested for their efficacy against aerosol challenge with subsp. *tularensis* SCHU S4 (Jia et al., 2009). BALB/c mice were sham-immunized, or primed-boosted i.d. with Lm Δ*actA* (vector control), rLm/iglC, rLm/katG (heterologous vaccines), or the LVS control, and challenged six weeks later with 1 × LD_50_ or 10 × LD_50_ aerosolized Schu S4_._ Mice primed-boosted with rLm/iglC had a greater survival rate (100 and 75% post-aerosol challenge with 1 × and 10 × LD_50_ SCHU S4, respectively) than sham-immunized mice or mice immunized with the vector control, and the survival rates were comparable to that of LVS-immunized mice (87.5%). Mice immunized with rLm/katG also showed greater survival than sham-immunized animals (Table 4, *Heterologous standalone vaccines*) (Jia et al., 2009). In subsequent studies, mice primed at Week 0 with LVS Δ*capB* or rLVS Δ*capB*/IglC (prime vaccines homologous to *F. tularenis*), boosted at Week 4 with heterologous vaccine rLm/iglC, and subsequently challenged at Week 10 with 10x LD_50_ aerosolized SCHU S4 had a significantly greater survival rate (75%) than sham-immunized mice (0%) (*p* < 0.0001), and the survival rate was greater than that of the LVS-immunized mice (62.5%) (Jia et al., 2013). Mice primed-boosted with rLVS ΔLPS/IglC—rLm/iglC (1 or 2 boosts) and subsequently challenged with 3 × or 10 × LD_50_ aerosolized SCHU S4 also had significantly greater survival rates than sham-immunized mice and mice immunized with the prime vaccine only (Table 4, *Homologous prime and heterologous boost vaccines*) (Jia et al., 2013).

**Table 4:** Live attenuated heterologous vaccine candidates: Protection against SCHU S4 respiratory challenge.

**Prime vaccine**	**Host strain**	**Prime route, dose (CFU)**	**Boost (vaccine, route)^a^**	**LVS control (route)^b^**	**Interval (days)^c^**	**SCHU S4 challenge route, dose (LD_50_)**	**% Survival post-challenge (MST, days)^d^**			**References**
							**Vaccine**	**LVS**	**Sham**	
**HETEROLOGOUS STANDALONE VACCINES**										
Lm Δ*actA* (LmV)	BALB/c	i.d., 10^6^	Yes (LmV × 1, i.d.)	Yes (i.d.)	42	Aerosol, 1	38	88	50	Jia et al., 2009
		i.d., 10^6^	Yes (LmV × 1, i.d.)	Yes (i.d.)	42	Aerosol, 10	38	88	0	Jia et al., 2009
rLm/iglC	BALB/c	i.d., 10^6^	Yes (rLm/iglC × 1, i.d.)	Yes (i.d.)	42	Aerosol, 1	100	88	50	Jia et al., 2009
		i.d., 10^6^	Yes (rLm/iglC × 1, i.d.)	Yes (i.d.)	42	Aerosol, 10	75	88	0 (6)	Jia et al., 2009
rLm/katG	BALB/c	i.d., 10^6^	Yes (rLm/katG × 1, i.d.)	Yes (i.d.)	42	Aerosol, 1	88	88	50	Jia et al., 2009
		i.d., 10^6^	Yes (rLm/katG × 1, i.d.)	Yes (i.d.)	42	Aerosol, 10	25	88	0 (6)	Jia et al., 2009
**HOMOLOGOUS PRIME AND HETEROLOGOUS BOOST VACCINES**										
LVS Δ*capB*	BALB/c	i.d., 10^6^	Yes (rLm/iglC × 1, i.d.)	Yes (i.d.)	42	Aerosol, 10	75 (19)	63 (17)	0 (5)	Jia et al., 2013
rLVS Δ*capB* /IglC	BALB/c	i.d., 10^6^	Yes (rLm/iglC × 1, i.d.)	Yes (i.d.)	42	Aerosol, 10	75 (20)	63 (17)	0 (5)	Jia et al., 2013
rLVS ΔLPS /IglC	BALB/c	i.d., 10^6^	Yes (rLm/iglC × 1, i.d.)	Yes (i.d.)	42	Aerosol, 3	63 (16)	100 (21)	0 (4)	Jia et al., 2013
rLVS ΔLPS /IglC	BALB/c	i.d., 10^6^	Yes (rLm/iglC × 2, i.d.)	Yes (i.d.)	42	Aerosol, 3	100 (21)	100 (21)	0 (4)	Jia et al., 2013
rLVS ΔLPS /IglC	BALB/c	i.d., 10^6^	Yes (rLm/iglC × 1, i.d.)	Yes (i.d.)	42	Aerosol, 10	38 (11)	100 (21)	0 (4)	Jia et al., 2013
rLVS ΔLPS /IglC	BALB/c	i.d., 10^6^	Yes (rLm/iglC × 2, i.d.)	Yes (i.d.)	42	Aerosol, 10	63 (15)	100 (21)	0 (4)	Jia et al., 2013

a *Boost: Heterologous standalone vaccines: mice were primed at Week 0 and boosted once (× 1) at Week 4 with Lm vector (LmV) or LmV expressing F. tularensis IglC (rLm/iglC) or KatG (rLm/katG); Homologous prime and heterologous boost vaccines: mice were primed at Week 0 with LVS ΔcapB or LVS ΔcapB overexpressing IglC (rLVS ΔcapB/IglC) or LVS ΔLPS overexpressing IglC (rLVS ΔLPS/IglC), and boosted with rLm/iglC once (× 1) at Week 4 or twice (× 2) at Weeks 3 and 6*.

b *LVS control: in heterologous standalone vaccine studies, LVS was given i.d. twice at Weeks 0 and 4; in homologous prime-heterologous boost studies, LVS was given once at Week 4*.

c *Interval: Time between the only or the last immunization and the challenge*.

d *Survival, % survival after challenge of mice immunized with the vaccine candidate (vaccine), LVS control (LVS), or no vaccine or PBS control (Sham). MST: Mean Survival Time*.

## Summary and discussion

The vaccines summarized here include deletional mutants of subsp. *holarctica* LVS or FSC200 (Table 1), *F. novicida* U112 (Table 2), and subsp. *tularensis* SCHU S4 (Table 3), and homologous LVS mutants overexpressing *F. tularensis* antigens and heterologous vectors expressing *F. tularensis* antigens (Table 4). The unlicensed LVS vaccine, the only vaccine available in the U.S.A., has shown substantial albeit incomplete efficacy in humans but retains residual toxicity. As noted, it would seem reasonable to expect that any vaccine warranting further consideration as a human vaccine satisfy the following two criteria: 1) the vaccine is safer than LVS; and 2) the vaccine provides protection equivalent to or greater than LVS against respiratory challenge with subsp. *tularensis* SCHU S4 in appropriate animal models. The data summarized here show that it is relatively easy to generate genetically defined mutants that are safer than LVS so as to meet the first criterion; however, it is difficult to control the balance between attenuation and protection so as to meet the second criterion simultaneously. Practically speaking, among the vaccine candidates summarized here, only a few meet both criteria. Often, the LVS-derived vaccine candidates need either prime-boost vaccination or intranasal administration in order to provide very high-level protective immunity.

With respect to the relative efficacies of vaccine candidates, in many cases, vaccines and challenge strains have been tested under different conditions, e.g., different preparations of vaccine and challenge strains; different routes of vaccine administration (mucosal vs. systemic); different immunization-challenge intervals (typically ranging from 3 to 6 weeks); different routes and doses of challenge strain (less virulent subsp. *holarctica* or *F. novicida* vs. highly virulent subsp. *tularensis* SCHU S4); and different animal models (mice, rats, rabbits, or non-human primates), making it difficult to compare their relative efficacies. Of course, head-to-head comparisons are the most reliable way to compare vaccine candidates, but except for comparisons with LVS—the only vaccine tested and shown protective in humans and effectively the current gold standard—this is rarely done. We summarize vaccines that were tested against respiratory challenge with SCHU S4 and compared with LVS for efficacy in Table 5 and discuss factors that affect vaccine efficacy below.

**Table 5:** Attenuation and protective efficacy against SCHU S4 respiratory challenge of *F. tularensis* vaccine candidates relative to LVS.

**Vaccine^a^**	**LD_50_ in mice (route, strain)**	**Efficacy ≥ LVS in mice against respiratory challenge with SCHU S4 (host strain)^b^**			**Vaccination—challenge interval (days) and other comments**	**References**
		**i.d**.	**i.n**.	**p.o**.		
LVS	~10^3^ (i.n. BALB/c) >10^7^ (i.d. BALB/c) >10^8^ (p.o., C3H/HeN)				Toxicity	Fortier et al., 1991; Jia et al., 2009; Conlan et al., 2010
**MORE ATTENUATED THAN LVS**						
LVS Δ*purMCD*	>10^6^ (i.n. BALB/c)		Yes with boost (BALB/c)		21	Pechous et al., 2008
LVS *sodB_*Ft*_*	>10^4^ (i.n. C57BL/6) >10^4^ (i.n. BALB/c)		Yes (C57BL/6)		21	Bakshi et al., 2006, 2008
FSC200 Δ*clpB*	>10^5^ (i.n. BALB/c)	Yes (BALB/c)			42; Less attenuated than LVS in SCID mice	Golovliov et al., 2013
LVS Δ*capB*	>10^7^ (i.n. BALB/c) >10^8^ (i.d. BALB/c)	No (BALB/c)	Yes (BALB/c)		42; Need i.n. vaccination	Jia et al., 2010, 2013, 2016
LVS Δ*capB* /IglA		Yes (BALB/c)			42	Jia et al., 2013
LVS Δ*capB* /IglC		Yes (BALB/c)			42	Jia et al., 2013
LVS Δ*capB* /IglABC		Yes (BALB/c)	Yes (BALB/c)		42	Jia et al., 2016, 2018
LVS::*wzy*	>10^7^ (i.n. BALB/c)		Yes (BALB/c)		28	Kim et al., 2012
LVS::*wbtA*	>10^7^ (i.n. BALB/c)		No (BALB/c)		28	Sebastian et al., 2007; Kim et al., 2012
Fn Δ*iglD*	>9.7 × 10^8^ (i.n. BALB/c)				30; No protection in BALB/c mice; relevant efficacy to LVS in mice and rats not known; efficacy in NHP equivalent to LVS	Chu et al., 2014
SCHU S4 Δ*purMCD*	>10^6^ (i.d. BALB/c) >10^6^ (i.n. BALB/c)		Yes with boost (BALB/c)		21; Single deletion	Pechous et al., 2008
SchuS4 Δ*gurBA*	>10^8^ (in. C57BL/6)				30; Efficacious in rabbits; single deletion	Reed et al., 2014; Santiago et al., 2015
SCHU S4 Δ*fipB*	>10^10^ (i.n. C57BL/6)				30; Less efficacious than LVS in rabbits; single deletion	Qin et al., 2009 Reed et al., 2014
SCHU S4 Δ*iglC*	>10^8^ (i.d. BALB/c)				42 or 63; Not efficacious; single deletion	Twine et al., 2005; Conlan et al., 2010
LVS Δ*capB* + rLm/iglC	>10^7^ (i.d. BALB/c) for both vaccines	Yes (BALB/c)			42	Jia et al., 2009, 2013
LVS Δ*capB*/Igl*C* + rLm/iglC	>10^7^ (i.d. BALB/c) for both vaccines	Yes (BALB/c)			42	Jia et al., 2009, 2013
**VIRULENCE EQUIVALENT TO LVS**						
SCHU S4 Δ*clpB*	>10^7^ (i.d. BALB/c)	Yes (BALB/c)		Yes (BALB/c)	42; Single deletion	Conlan et al., 2010; Shen et al., 2010
SCHU S4 Δ*clpB*Δ*capB*	>10^7^ (i.d. BALB/c)	Yes (BALB/c)			42; Survival ≤ 20%	Golovliov et al., 2013
**LESS ATTENUATED THAN LVS**						
SCHU S4 ΔFTT0918	~10^5^ (i.d. BALB/c)	Yes (BALB/c)			42; More virulent than LVS in mice	Twine et al., 2005
SCHU S4 ΔFTT0918 Δ*capB*	≥ 10^7^ (i.d. BALB/c) ~10^3^ (i.d. C3H/HeN)	Yes (BALB/c)		Yes (C3H/HeN)	42; Protection in C3H mice ≥ LVS with i.d. prime and oral boost; more virulent than LVS in C3H mice	Conlan et al., 2010
**VIRULENCE NOT COMPARED WITH LVS**						
SchuS4 Δ*aroD*	Not available				21; More protective than LVS in rabbits; single deletion	Reed et al., 2014

a *Vaccine: Only vaccines that were tested against subsp. tularensis (Type A) respiratory challenge and compared with LVS are listed*.

b *Efficacy relative to LVS: Percentage survival was used to compare the protective efficacy against respiratory challenge with a subsp. tularensis Type A strain between animals immunized with the vaccine candidate and animals immunized with LVS. Statistical significance was not available from some of the reported studies*.

### Vaccine and challenge strain stock preparation

As summarized in Tables 1–4, some studies used LVS as a positive control to evaluate the efficacy of various vaccines against respiratory challenge with SCHU S4 strains. It is interesting that while the median/mean survival time for unvaccinated or sham-immunized mice fell between 4 and 6 days post-respiratory SCHU S4 challenge in most studies, the immunity induced by LVS varied significantly in these studies. Of note, some vaccine strains were produced on solid agar while others in broth medium. Eigelsbach et al. conducted a study on virulence and immunogenicity of live vaccine strains. The study showed that LVS harvested from modified casein partial hydrolysate medium (MCPH) appeared more virulent and immunogenic in mice than LVS harvested from glucose cysteine hemin agar (GCHA); however, LVS prepared from GCHA and MCPH induced comparable protection in guinea pigs (Eigelsbach and Downs, 1961). While it is impossible to compare vaccines among different studies, it would be helpful to include LVS prepared using a standardized method as a positive control in efficacy studies.

### Mucosal vs. systemic route of vaccine administration

As noted above, several live attenuated vaccine candidates induce more potent protective immunity against respiratory challenge with subsp. *tularensis* SCHU S4 when administered by the mucosal respiratory route (e.g., i.n., aerosol, or intratrachea), or by the mucosal oral route than by a traditional systemic route (i.d., s.c., or i.p.) (Hornick and Eigelsbach, 1966; Conlan et al., 2005; Wu et al., 2005; Pechous et al., 2008; Jia et al., 2010, 2013), with a few exceptions (Rockx-Brouwer et al., 2012). In one study, an alternative mucosal (i.e., oral) route for delivery of *F. novicida-*derived vaccine candidates showed greater protective immunity than the i.n. route against respiratory challenge with the virulent SCHU S4 strain (Cong et al., 2009). The i.n. route raises some additional safety concerns; that said, the current live attenuated flu vaccine administered i.n. has an excellent safety record (Pavot et al., 2012).

### Interval between vaccination and challenge

As summarized in Tables 1–4, intervals between the last or the only vaccination and challenge ranged between 3 and 6 weeks in different studies. Eigelsbach and Downs investigated the effect of the vaccination-challenge interval on the immunity of LVS in mice and guinea pigs (Eigelsbach and Downs, 1961). Albino mice (Webster) vaccinated with LVS s.c. 15–30 days prior to challenge with 10^3^ CFU of SCHU S4 s.c. had a higher survival rate (68–88%) than mice vaccinated 60 days prior to challenge (49–56%). This difference was appreciably greater in guinea pigs. Guinea pigs (Harley) vaccinated 15 days prior to challenge had 45% survival vs. 5% survival for those vaccinated 30 days prior to challenge. LVS administered i.d. or i.n. to mice was cleared from all the infected organs by 3 weeks (Jia et al., 2010). Thus, it would be helpful to challenge animals at a longer interval, i.e., more than 3 weeks, to minimize the effect of non-specific immunity and to allow evaluation of various vaccines under more rigorous conditions.

### Hypo- and hyper- attenuation of vaccines

Some mutants of subsp. *holactica* LVS (i.e., LVS Δ*purMCD*, LVS sodB_κ_, LVS Δ*capB*, LVS Δ*capB*/*iglA*, LVS Δ*capB*/iglABC, and LVS Δ*wzy*) and subsp. *tularensis* SCHU S4 (SCHU S4Δ*purMCD*, SCHU S4Δ*clpB*, SCHU S4Δ*clpB*Δ*capB*) show significant attenuation and promising protective immunity against respiratory challenge in a murine model (Table 5) (Bakshi et al., 2006, 2008; Pechous et al., 2008; Qin et al., 2009; Sebastian et al., 2009; Jia et al., 2010, 2013, 2016; Shen et al., 2010; Kim et al., 2012; Rockx-Brouwer et al., 2012; Twine et al., 2012; Golovliov et al., 2013; Marohn and Barry, 2013; Straskova et al., 2015); other mutants, however, are either hypo- or hyper- attenuated, rendering them either poorly immunogenic or too virulent to use. LVS-derived vaccine candidates have deletions of at least three major virulence genes including two that were lost in the generation of the parental LVS strain (Salomonsson et al., 2009). Most immunoprotective SCHU S4 mutants, however, are single deletional mutants, raising the concern that they are only one mutation away from reversion to virulence, as seen with viral pathogens (Jia et al., 1999; Zhou et al., 2016). Therefore, a second major attenuating deletion is generally thought necessary—this typically markedly reduces their capacity to induce protective immunity. For example, the SCHU S4 Δ*clpB* mutant is highly attenuated (LD_50_ > 10^7^ i.d.) and protective against respiratory challenge with ≤ 100 CFU SCHU S4 (40–100% of immunized BALB/c mice survived challenge)—more protective than LVS. However, introduction of a second major attenuating deletion in various genes (*pmrA, relA, capB, wbtC, ggt*, or *fupA*) significantly reduces its capacity to induce protective immunity against SCHU S4 challenge (<20% of mice immunized with the double deletional mutants survived SCHU S4 challenge)(Table 3, Heat shock protein mutants) (Golovliov et al., 2013). Another example is a SCHU S4 mutant with a single deletion in FTT0369c or FTT1676; these show significant protection against respiratory challenge with SCHU S4; however, a double deletion in both FTT0369c and FTT1676 significantly reduces the vaccine's capacity to induce protective immunity (Table 3, *Other mutants*) (Rockx-Brouwer et al., 2012).

### Vaccine genetic background

Vaccines have been generated in the background of subsp. *holarctica*, subsp. *tularensis, F*. *novicida*, and heterologous vectors. The subsp. *holarctica* LVS strain is the only vaccine tested and shown efficacious in humans against virulent SCHU S4 challenge; however, its residual toxicity in humans and unknown attenuation mechanism may have presented obstacles to its licensure. LVS-derived vaccines are safer than LVS and retain the large antigen pool that might be required for protection against heterologous challenge with the virulent *subsp. tularensis* strain. However, a booster or intranasal vaccination route are generally required for enhanced vaccine-induced protection (Table 1). *F. novicida* is less virulent than LVS in mice, guinea pigs, rabbits and Fisher rats, and rarely infects humans (so far only 12 cases have been documented) (Kingry and Petersen, 2014). However, in contrast to LVS, *F. novicida* differs from *F. tularensis* in the mechanism of pathogenicity; they differ in cell surface structure, means of cellular entry, types of cells infected *in vivo*, and ability to evade host immune responses (Kingry and Petersen, 2014). The *F. novicida* parental strain and its derivatives have not shown efficacy against respiratory challenge with SCHU S4 in mice and guinea pigs by i.d. or i.n. route; the protective immunity induced by the i.t. or oral route against i.t. challenge with SCHU S4 in Fisher rats was not compared with that of LVS (Table 2). The subsp. *tularensis*-derived vaccines are generally more efficacious than LVS against homologous challenge with SCHU S4. However, this may be true only for SCHU S4-derived vaccines with a single deletion. A second major attenuating deletion significantly reduces vaccine efficacy (Table 3). The vaccines constructed using a heterologous vector (i.e., *Listeria monocytogenes*) have a much more limited antigen pool, need multiple vaccinations, and are less efficacious than LVS (Table 4).

The impact of vaccine genetic background on vaccine efficacy is also evident in comparisons of vaccines derived from different subspecies but comprising the same deletional gene mutation. For example, some gene deletional mutants in the LVS background are both highly attenuated and able to induce protective immunity against SCHU S4 challenge, but this may not be true of the same deletion in the *F. novicida* or SCHU S4 background. An LVS mutant with a deletion in *purMCD*, LVS Δ*purMCD*, is both highly attenuated and able to induce protection against virulent SCHU S4 i.n. challenge; protective immunity is similar to that induced by LVS i.n. vaccination. However, an attenuated SCHU S4 with the same deletion, SCHU S4 Δ*purMCD*, provides limited protection against SCHU S4 challenge, less than that induced by LVS vaccination (Pechous et al., 2006, 2008). Attenuated *F. novicida* mutants with similar deletions, Δ*purCD* and Δ*purM*, are not able to induce protection against the homologous wild-type strain challenge (Tempel et al., 2006; Quarry et al., 2007). The same findings have been reported for *guaA, guaB*, and *guaBA* mutants (Santiago et al., 2009, 2015). In contrast, *clpB* deletional mutants in the subsp. *tularensis* (SCHU S4) background induced full protection against respiratory challenge with the parent SCHU S4; a mutant with the same deletion in LVS showed no protection (Golovliov et al., 2013). Hence, the vaccine's genetic background is an important determinant of the vaccine's attenuation and protective efficacy.

### Animal model

Various animal models—mice, rats, rabbits, guinea pigs, and non-human primates—have been used to study tularemia vaccine efficacy as reviewed recently by Elkins et al. (2016); however, they differ in their sensitivity to the highly virulent SCHU S4 strain and in vaccine-induced protective immunity against SCHU S4 challenge. Mice, guinea pigs, rabbits, and primates are more sensitive to SCHU S4 than rats (White rats and Fisher rats). The LD_50_ for SCHU S4 by a systemic route (s.c. or i.d.) is 1 CFU in mice, 1 CFU in guinea pigs, 1 CFU in rabbits, and >10^8^ CFU (s.c.) in White rats; the LD_50_ of SCHU S4 by the respiratory route is 1-3 CFU in mice (i.n.), 1 CFU in cynomolgus macaques (aerosol), and >5 × 10^2^ CFU in Fisher rats (intratreacheal); in humans, as few as 10–50 CFU SCHU S4 can cause clinical tularemia (Eigelsbach et al., 1951; McCrumb, 1961; Saslaw et al., 1961a,b; Schricker et al., 1972; Kostiala et al., 1975; Jemski, 1981; Conlan et al., 2003; Barker and Klose, 2007; Lyons and Wu, 2007; Raymond and Conlan, 2009; Wu et al., 2009; Ray et al., 2010; Chu et al., 2014; Kingry and Petersen, 2014; Hutt et al., 2017; Nguyen et al., 2017). Mice, rats, guinea pigs, and primates also differ in the degree of vaccine-induced protective immunity against virulent SCHU S4 challenge. Mice, guinea pigs, and humans vaccinated with the Foshay-killed vaccine were not protected against aerosol challenge with SCHU S4, while White rats were protected (Lyons and Wu, 2007). Furthermore, different strains within species show substantial variability in their susceptibility to challenge and the degree of vaccine-induced protection. LVS-immunized BALB/c mice are more resistant than LVS-immunized C57BL/6 mice to low dose aerosol or high dose i.d. challenge with virulent subsp. *tularensis* FSC 033 strain (Chen et al., 2003); however, C57BL/6 mice clear subsp. *tularensis* SCHU S4 infection more rapidly (Fritz et al., 2014). White rats and Fisher rats are more resistant to SCHU S4 challenge than Sprague–Dawley rats (Lyons and Wu, 2007; Raymond and Conlan, 2009). African green monkeys and cynomolgus monkeys are more sensitive to SCHU S4 aerosol challenge than Rhesus monkeys, with an aerosol lethal dose of 40, 32, and 2.8 × 10^5^ CFU, respectively (Glynn et al., 2015). In addition, recent studies show that vaccines inducing strong protective immunity in one animal model may not do so in another model. For example, *guaBA, aroD*, and *fipB* (FTT1103) mutants have been tested in both mice and rabbits (Qin et al., 2009; Santiago et al., 2009, 2015; Reed et al., 2014). While the SCHU S4 Δ*guaB* and Δ*guaA* mutants, administered via scarification, induce partial protective immunity against respiratory SCHU S4 challenge in the New Zealand rabbit model, they do not do so in C57BL mice (Reed et al., 2014; Santiago et al., 2015). In contrast, the SCHU S4 Δ*fibB* (FTT1103) mutant, while fully protective in C57BL mice and partially protective in BALB/c mice, showed no protection against SCHU S4 challenge in the rabbit model (Qin et al., 2009; Reed et al., 2014). Other reports have reported differences in protective immunity conferred by the same vaccine in the murine and Fisher rat models (Cong et al., 2009; Signarovitz et al., 2012; Chu et al., 2014). These findings highlight the impact of animal model on the outcomes of preclinical vaccine efficacy studies.

The low natural incidence of tularemia renders field trials of efficacy unfeasible, and ethical considerations make it unlikely that tularemia vaccines will ever again be tested for efficacy in human challenge studies. In such situations, the FDA has promulgated the Animal Rule, whereby a drug or vaccine may be licensed on the basis of efficacy in relevant animal models. That raises the question as to which animal models are most relevant. If a vaccine is ineffective in the highly sensitive mouse model, are efficacy studies in the relatively resistant Fisher rat model an acceptable substitute? Proponents of this rat model would argue that the susceptibility of the Fisher rat to various *F. tularensis* strains more closely approximates that of humans than the mouse. Be it as it may, from the standpoint of efficacy, a vaccine that is efficacious in the most sensitive animal models would seem to be a more reliable one for humans, who likely have varying susceptibility to infection depending upon numerous host variables, than a vaccine efficacious only in relatively resistant models.

## Conclusion

Since 2001, several promising live attenuated vaccine candidates have been developed that meet what would seem to be minimal criteria for a new human vaccine—safety greater than LVS and protective efficacy equivalent to or greater than LVS against respiratory challenge with subsp. *tularensis* SCHU S4 in appropriate animal models (Table 5). Some of these vaccines additionally demonstrate efficacy comparable to or greater than that of LVS against SCHU S4 respiratory challenge in the demanding mouse model. Vaccines meeting this higher standard include LVS mutants (LVS Δ*purMCD*, LVS *sodB*_*Ft*_, LVS Δ*capB*, and LVS::*wzy*), another *F. tularensis* type B mutant (FSC200Δ*clpB*), LVS Δ*capB* overexpressing *F. tularensis* T6SS proteins (LVS Δ*capB*/IglA, IglC or IglABC), a LVS Δ*capB*-rLm/IglC heterologous prime-boost vaccine, and a single deletional SCHU S4 mutant (Δ*purMCD*). Another single deletional SCHU S4 mutant, SCHU S4 Δ*clpB*, and a double deletional SCHU S4 mutant, SCHU S4Δ*clpB*Δ*capB* demonstrates efficacy greater than LVS but its attenuation is equivalent to LVS. That these SCHU S4 mutants contain only one major attenuating deletion raises a safety concern, namely reversion to virulence. A second major attenuating deletion would alleviate this concern, but retaining efficacy upon the introduction of a second major attenuating mutation has been a major challenge for single deletional SCHU S4 mutant vaccines. The efficacy of the new vaccines has typically been greatest when administered by the intranasal or another respiratory route, but administering vaccines by this route raises additional safety issues. Nevertheless, vaccines that are safer than LVS and at least as efficacious, including by the i.d. route, have been developed, and they are promising candidates for going forward into more advanced animal studies such as in the non-human primate model and human safety trials.

## Author contributions

QJ and MAH wrote the article. The comments in this article represent the opinions of both QJ and MAH.

### Conflict of interest statement

The authors declare inventors on patents describing tularemia vaccines that are owned by UCLA.

## Footnotes

^1^Centers for Disease Control and Prevention, Tularemia Prevention, https://www.cdc.gov/tularemia/prevention/index.html, October 26, 2015.

^2^U. S. Food and Drug Administration, Vaccines Licensed for Use in the United States, https://www.fda.gov/BiologicsBloodVaccines/Vaccines/ApprovedProducts/ucm093833.htm, 02/14/2018.

^3^European Medicines Agency. EMA/CHMP Guidance document on use of medicinal products for the treatment and prophylaxis of biological agents that might be used as weapons of bioterrorism, http://www.ema.europa.eu/docs/en_GB/document_library/Regulatory_and_procedural_guideline/2010/01/WC500049399.pdf, 18 November 2014.

## References

[B1] Alibek K. (1999). Biohazard: The Chilling True Story of the Largest Covert Biological Weapons Program in the World, Told From the Inside by the Man Who Ran It. New York, NY: Random House, Inc.

[B2] Bakshi C. S. Malik M. Mahawar M. Kirimanjeswara G. S. Hazlett K. R. Palmer L. E.. (2008). An improved vaccine for prevention of respiratory tularemia caused by *Francisella tularensis* SchuS4 strain. Vaccine 26, 5276–5288. 10.1016/j.vaccine.2008.07.051 18692537 PMC2652725

[B3] Bakshi C. S. Malik M. Regan K. Melendez J. A. Metzger D. W. Pavlov V. M.. (2006). Superoxide dismutase B gene (sodB)-deficient mutants of *Francisella tularensis* demonstrate hypersensitivity to oxidative stress and attenuated virulence. J. Bacteriol. 188, 6443–6448. 10.1128/JB.00266-06 16923916 PMC1595384

[B4] Banik S. Mansour A. A. Suresh R. V. Wykoff-Clary S. Malik M. McCormick A. A.. (2015). Development of a multivalent subunit vaccine against tularemia using Tobacco Mosaic Virus (TMV) based delivery system. PLoS ONE 10:e0130858. 10.1371/journal.pone.0130858 26098553 PMC4476615

[B5] Barker J. R. Klose K. E. (2007). Molecular and genetic basis of pathogenesis in *Francisella tularensis*. Ann. N. Y. Acad. Sci. 1105, 138–59. 10.1196/annals.1409.010 17395737

[B6] Barrigan L. M. Tuladhar S. Brunton J. C. Woolard M. D. Chen C. J. Saini D.. (2013). Infection with *Francisella tularensis* LVS clpB leads to an altered yet protective immune response. Infect. Immun. 81, 2028–2042. 10.1128/IAI.00207-13 23529616 PMC3676025

[B7] Bernard K. Tessier S. Winstanley J. Chang D. Borczyk A. (1994). Early recognition of atypical *Francisella tularensis* strains lacking a cysteine requirement. J. Clin. Microbiol. 32, 551–553. 8150973 10.1128/jcm.32.2.551-553.1994 PMC263075

[B8] Bosio C. M. Bielefeldt-Ohmann H. Belisle J. T. (2007). Active suppression of the pulmonary immune response by *Francisella tularensis* Schu4. J. Immunol. 178, 4538–4547. 10.4049/jimmunol.178.7.4538 17372012

[B9] Buchan B. W. McCaffrey R. L. Lindemann S. R. Allen L. A. H. Jones B. D. (2009). Identification of migR, a regulatory element of the *Francisella tularensis* live vaccine strain iglABCD virulence operon required for normal replication and trafficking in macrophages. Infect. Immun. 77, 2517–2529. 10.1128/IAI.00229-09 19349423 PMC2687360

[B10] Chen W. Shen H. Webb A. KuoLee R. Conlan J. W. (2003). Tularemia in BALB/c and C57BL/6 mice vaccinated with *Francisella tularensis* LVS and challenged intradermally, or by aerosol with virulent isolates of the pathogen: protection varies depending on pathogen virulence, route of exposure, and host genetic background. Vaccine 21, 3690–3700. 10.1016/S0264-410X(03)00386-4 12922099

[B11] Christopher G. W. Cieslak T. J. Pavlin J. A. Eitzen E. M. Jr. (1997). Biological warfare. A historical perspective. JAMA 278, 412–417. 9244333

[B12] Chu P. Cunningham A. L. Yu J. J. Nguyen J. Q. Barker J. R. Lyons C. R.. (2014). Live attenuated *Francisella novicida* vaccine protects against *Francisella tularensis* pulmonary challenge in rats and non-human primates. PLoS Pathog. 10:e1004439. 10.1371/journal.ppat.1004439 25340543 PMC4207810

[B13] Clemens D. L. Ge P. Lee B. Y. Horwitz M. A. Zhou Z. H. (2015). Atomic structure of T6SS reveals interlaced array essential to function. Cell 160, 940–951. 10.1016/j.cell.2015.02.005 25723168 PMC4351867

[B14] Clemens D. L. Horwitz M. A. (2007). Uptake and intracellular fate of *Francisella tularensis* in human macrophages. Ann. N. Y. Acad. Sci. 1105, 160–186. 10.1196/annals.1409.001 17435118

[B15] Clemens D. L. Lee B. Y. Horwitz M. A. (2004). Virulent and avirulent strains of *Francisella tularensis* prevent acidification and maturation of their phagosomes and escape into the cytoplasm in human macrophages. Infect. Immun. 72, 3204–3217. 10.1128/IAI.72.6.3204-3217.2004 15155622 PMC415696

[B16] Clemens D. L. Lee B. Y. Horwitz M. A. (2005). *Francisella tularensis* enters macrophages via a novel process involving pseudopod loops. Infect. Immun. 73, 5892–5902. 10.1128/IAI.73.9.5892-5902.2005 16113308 PMC1231130

[B17] Cole L. E. Yang Y. Elkins K. L. Fernandez E. T. Qureshi N. Shlomchik M. J.. (2009). Antigen-specific B-1a antibodies induced by *Francisella tularensis* LPS provide long-term protection against F. tularensis LVS challenge. Proc. Natl. Acad. Sci. U.S.A. 106, 4343–4348. 10.1073/pnas.0813411106 19251656 PMC2657382

[B18] Cong Y. Yu J. J. Guentzel M. N. Berton M. T. Seshu J. Klose K. E.. (2009). Vaccination with a defined *Francisella tularensis* subsp. novicida pathogenicity island mutant (DeltaiglB) induces protective immunity against homotypic and heterotypic challenge. Vaccine 27, 5554–5561. 10.1016/j.vaccine.2009.07.034 19651173 PMC2752818

[B19] Conlan J. W. (2011). Tularemia vaccines: recent developments and remaining hurdles. Future Microbiol. 6, 391–405. 10.2217/fmb.11.22 21526941

[B20] Conlan J. W. Chen W. Shen H. Webb A. KuoLee R. (2003). Experimental tularemia in mice challenged by aerosol or intradermally with virulent strains of *Francisella tularensis*: bacteriologic and histopathologic studies. Microb. Pathog. 34, 239–248. 10.1016/S0882-4010(03)00046-9 12732472

[B21] Conlan J. W. Shen H. Golovliov I. Zingmark C. Oyston P. C. Chen W.. (2010). Differential ability of novel attenuated targeted deletion mutants of *Francisella tularensis* subspecies tularensis strain SCHU S4 to protect mice against aerosol challenge with virulent bacteria: effects of host background and route of immunization. Vaccine 28, 1824–1831. 10.1016/j.vaccine.2009.12.001 20018266 PMC2822029

[B22] Conlan J. W. Shen H. Webb A. Perry M. B. (2002). Mice vaccinated with the O-antigen of *Francisella tularensis* LVS lipopolysaccharide conjugated to bovine serum albumin develop varying degrees of protective immunity against systemic or aerosol challenge with virulent type A and type B strains of the pathogen. Vaccine 20, 3465–3471. 10.1016/S0264-410X(02)00345-6 12297391

[B23] Conlan W. J. Shen H. Kuolee R. Zhao X. Chen W. (2005). Aerosol-, but not intradermal-immunization with the live vaccine strain of *Francisella tularensis* protects mice against subsequent aerosol challenge with a highly virulent type A strain of the pathogen by an alphabeta T cell- and interferon gamma- dependent mechanism. Vaccine 23, 2477–2485. 10.1016/j.vaccine.2004.10.034 15752834

[B24] Cunningham A. L. Dang K. M. Yu J. J. Guentzel M. N. Heidner H. W. Klose K. E.. (2014). Enhancement of vaccine efficacy by expression of a TLR5 ligand in the defined live attenuated *Francisella tularensis* subsp. novicida strain U112DeltaiglB::fljB. Vaccine 32, 5234–5240. 10.1016/j.vaccine.2014.07.038 25050972 PMC4140432

[B25] Dennis D. T. Inglesby T. V. Henderson D. A. Bartlett J. G. Ascher M. S. Eitzen E.. (2001). Tularemia as a biological weapon: medical and public health management. JAMA 285, 2763–2773. 10.1001/jama.285.21.2763 11386933

[B26] Eigelsbach H. T. Braun W. Herring R. D. (1951). Studies on the variation of Bacterium tularense. J. Bacteriol. 61, 557–569. 14832199 10.1128/jb.61.5.557-569.1951 PMC386045

[B27] Eigelsbach H. T. Downs C. M. (1961). Prophylactic effectiveness of live and killed tularemia vaccines. I. Production of vaccine and evaluation in the white mouse and guinea pig. J. Immunol. 87, 415–425. 13889609

[B28] Elkins K. L. Cowley S. C. Bosio C. M. (2007). Innate and adaptive immunity to Francisella. Ann. N. Y. Acad. Sci. 1105, 284–324. 10.1196/annals.1409.014 17468235

[B29] Elkins K. L. Kurtz S. L. De Pascalis R. (2016). Progress, challenges, and opportunities in Francisella vaccine development. Expert Rev. Vaccines 15, 1183–1196. 10.1586/14760584.2016.1170601 27010448

[B30] El Sahly H. M. Atmar R. L. Patel S. M. Wells J. M. Cate T. Ho M.. (2009). Safety, reactogenicity and immunogenicity of *Francisella tularensis* live vaccine strain in humans. Vaccine 27, 4905–4911. 10.1016/j.vaccine.2009.06.036 19567246 PMC2726995

[B31] Ericsson M. Kroca M. Johansson T. Sjostedt A. Tarnvik A. (2001). Long-lasting recall response of CD4+ and CD8+ alphabeta T cells, but not gammadelta T cells, to heat shock proteins of *Francisella tularensis*. Scand J. Infect. Dis. 33, 145–152. 10.1080/003655401750065562 11233852

[B32] Ericsson M. Sandstrom G. Sjostedt A. Tarnvik A. (1994). Persistence of cell-mediated immunity and decline of humoral immunity to the intracellular bacterium *Francisella tularensis* 25 years after natural infection. J. Infect. Dis. 170, 110–114. 10.1093/infdis/170.1.110 8014484

[B33] Forsman M. Sandstrom G. Sjostedt A. (1994). Analysis of 16S ribosomal DNA sequences of Francisella strains and utilization for determination of the phylogeny of the genus and for identification of strains by PCR. Int. J. Syst. Bacteriol. 44, 38–46. 10.1099/00207713-44-1-38 8123561

[B34] Fortier A. H. Slayter M. V. Ziemba R. Meltzer M. S. Nacy C. A. (1991). Live vaccine strain of *Francisella tularensis*: infection and immunity in mice. Infect. Immun. 59, 2922–2928. 1879918 10.1128/iai.59.9.2922-2928.1991 PMC258114

[B35] Foshay L. (1932). Prophylactic vaccination against tularemia. Am. J. Clin. Pathol. 2, 7–10.

[B36] Foshay L. Hesselbrock W. H. Wittenberg H. J. Rodenberg A. H. (1942). Vaccine Prophylaxis against Tularemia in Man. Am. J. Public Health Nation's Health 32, 1131–1145. 18015689 10.2105/ajph.32.10.1131 PMC1527181

[B37] Francis E. (1925). Tularemia. JAMA 84, 1243–1250. 10.1001/jama.1925.02660430001001 6358564

[B38] Francis E. (1928). A summary of present knowledge of tularaemia. J. Clin. Endocrinol. 7, 411–432.

[B39] Fritz D. L. England M. J. Miller L. Waag D. M. (2014). Mouse models of aerosol-acquired tularemia caused by *Francisella tularensis* types A and B. Comp. Med. 64, 341–50. 25402174 PMC4236782

[B40] Fulop M. Mastroeni P. Green M. Titball R. W. (2001). Role of antibody to lipopolysaccharide in protection against low- and high-virulence strains of *Francisella tularensis*. Vaccine 19, 4465–4472. 10.1016/S0264-410X(01)00189-X 11483272

[B41] Gillette D. D. Curry H. M. Cremer T. Ravneberg D. Fatehchand K. Shah P. A.. (2014). Virulent Type A *Francisella tularensis* actively suppresses cytokine responses in human monocytes. Front. Cell. Infect. Microbiol. 4:45. 10.3389/fcimb.2014.00045 24783062 PMC3988375

[B42] Glynn A. R. Alves D. A. Frick O. Erwin-Cohen R. Porter A. Norris S.. (2015). Comparison of experimental respiratory tularemia in three nonhuman primate species. Comp. Immunol. Microbiol. Infect. Dis. 39, 13–24. 10.1016/j.cimid.2015.01.003 25766142 PMC4397973

[B43] Golovliov I. Ericsson M. Akerblom L. Sandstrom G. Tarnvik A. Sjostedt A. (1995). Adjuvanticity of ISCOMs incorporating a T cell-reactive lipoprotein of the facultative intracellular pathogen *Francisella tularensis*. Vaccine 13, 261–267. 10.1016/0264-410X(95)93311-V 7631511

[B44] Golovliov I. Twine S. M. Shen H. Sjostedt A. Conlan W. (2013). A DeltaclpB mutant of *Francisella tularensis* subspecies holarctica strain, FSC200, is a more effective live vaccine than F. tularensis LVS in a mouse respiratory challenge model of tularemia. PLoS ONE 8:e78671. 10.1371/journal.pone.0078671 24236032 PMC3827231

[B45] Harris S. (1992). Japanese biological warfare research on humans: a case study of microbiology and ethics. Ann. N. Y. Acad. Sci. 666, 21–52. 10.1111/j.1749-6632.1992.tb38021.x 1297279

[B46] Honn M. Lindgren H. Bharath G. K. Sjostedt A. (2017). Lack of OxyR and KatG results in extreme susceptibility of *Francisella tularensis* LVS to oxidative stress and marked attenuation *in vivo*. Front. Cell. Infect. Microbiol. 7:14. 10.3389/fcimb.2017.00014 28174696 PMC5258697

[B47] Honn M. Lindgren H. Sjostedt A. (2012). The role of MglA for adaptation to oxidative stress of *Francisella tularensis* LVS. BMC Microbiol. 12:14. 10.1186/1471-2180-12-14 22264342 PMC3305382

[B48] Hornick R. B. Dawkins A. T. Eigelsbach H. T. Tulis J. J. (1966). Oral tularemia vaccine in man. Antimicrob. Agents Chemother. 6, 11–14. 4860768 10.1128/AAC.6.1.11

[B49] Hornick R. B. Eigelsbach H. T. (1966). Aerogenic immunization of man with live Tularemia vaccine. Bacteriol. Rev. 30, 532–538. 5917334 10.1128/br.30.3.532-538.1966 PMC378235

[B50] Huber B. Escudero R. Busse H. J. Seibold E. Scholz H. C. Anda P.. (2010). Description of Francisella hispaniensis sp nov., isolated from human blood, reclassification of Francisella novicida (Larson et al. 1955) Olsufiev et al. 1959 as *Francisella tularensis* subsp novicida comb. nov and emended description of the genus Francisella. Int. J. Syst. Evol. Micr. 60, 1887–1896. 10.1099/ijs.0.015941-0 19783615

[B51] Hutt J. A. Lovchik J. A. Dekonenko A. Hahn A. C. Wu T. H. (2017). The natural history of pneumonic tularemia in female fischer 344 rats after inhalational exposure to aerosolized *Francisella tularensis* subspecies tularensis strain SCHU S4. Am. J. Pathol. 187, 252–267. 10.1016/j.ajpath.2016.09.021 27939130 PMC5389373

[B52] Ireland P. M. LeButt H. Thomas R. M. Oyston P. C. (2011). A *Francisella tularensis* SCHU S4 mutant deficient in gamma-glutamyltransferase activity induces protective immunity: characterization of an attenuated vaccine candidate. Microbiology 157, 3172–3179. 10.1099/mic.0.052902-0 21852349

[B53] Jemski J. V. (1981). Respiratory tularemia: comparison of selected routes of vaccination in Fischer 344 rats. Infect. Immun. 34, 766–772. 7333669 10.1128/iai.34.3.766-772.1981 PMC350937

[B54] Jia Q. Bowen R. Dillon B. J. Masleša-Galić S. Chang B. T. Kaidi A. C.. (2018). Single vector platform vaccine protects against lethal respiratory challenge with Tier 1 select agents of anthrax, plague, and tularemia. Sci Rep. 8:7009. 10.1038/s41598-018-24581-y 29725025 PMC5934503

[B55] Jia Q. Bowen R. Lee B. Y. Dillon B. J. Maslesa-Galic S. Horwitz M. A. (2016). *Francisella tularensis* Live Vaccine Strain deficient in capB and overexpressing the fusion protein of IglA, IglB, and IglC from the bfr promoter induces improved protection against *F. tularensis* respiratory challenge. Vaccine 34, 4969–78. 10.1016/j.vaccine.2016.08.041 27577555 PMC5028307

[B56] Jia Q. Bowen R. Sahakian J. Dillon B. J. Horwitz M. A. (2013). A heterologous prime-boost vaccination strategy comprising the *Francisella tularensis* live vaccine strain capB mutant and recombinant attenuated *Listeria monocytogenes* expressing F. tularensis IglC induces potent protective immunity in mice against virulent *F. tularensis* aerosol challenge. Infect. Immun. 81, 1550–1561. 10.1128/IAI.01013-12 23439306 PMC3647989

[B57] Jia Q. Lee B. Y. Bowen R. Dillon B. J. Som S. M. Horwitz M. A. (2010). A *Francisella tularensis* live vaccine strain (LVS) mutant with a deletion in capB, encoding a putative capsular biosynthesis protein, is significantly more attenuated than LVS yet induces potent protective immunity in mice against *F. tularensis* challenge. Infect. Immun. 78, 4341–4355. 10.1128/IAI.00192-10 20643859 PMC2950357

[B58] Jia Q. Lee B. Y. Clemens D. L. Bowen R. A. Horwitz M. A. (2009). Recombinant attenuated *Listeria monocytogenes* vaccine expressing *Francisella tularensis* IglC induces protection in mice against aerosolized Type A *F. tularensis*. Vaccine 27, 1216–1229. 10.1016/j.vaccine.2008.12.014 19126421 PMC2654553

[B59] Jia Q. Ohka S. Iwasaki K. Tohyama K. Nomoto A. (1999). Isolation and molecular characterization of a poliovirus type 1 mutant that replicates in the spinal cords of mice. J. Virol. 73, 6041–6047. 10364356 10.1128/jvi.73.7.6041-6047.1999 PMC112665

[B60] Johansson A. Celli J. Conlan W. Elkins K. L. Forsman M. Keim P. S.. (2010). Objections to the transfer of Francisella novicida to the subspecies rank of *Francisella tularensis*. Int. J. Syst. Evol. Micr. 60, 1717–1718. 10.1099/ijs.0.022830-0 20688748 PMC7442299

[B61] Kadull P. J. Reames H. R. Coriell L. L. Foshay L. (1950). Studies on tularemia. V. Immunization of man. J. Immunol. 65, 425–435. 14794905

[B62] Kanistanon D. Hajjar A. M. Pelletier M. R. Gallagher L. A. Kalhorn T. Shaffer S. A.. (2008). A Francisella mutant in lipid A carbohydrate modification elicits protective immunity. PLoS Pathog. 4:e24. 10.1371/journal.ppat.0040024 18266468 PMC2233673

[B63] Karttunen R. Andersson G. Ekre H. P. Juutinen K. Surcel H. M. Syrjala H. (1987). Interleukin 2 and gamma interferon production, interleukin 2 receptor expression, and DNA synthesis induced by tularemia antigen *in vitro* after natural infection or vaccination. J. Clin. Microbiol. 25, 1074–1078. 3110207 10.1128/jcm.25.6.1074-1078.1987 PMC269139

[B64] Kaur R. Chen S. Arevalo M. T. Xu Q. Chen Y. Zeng M. (2012). Protective immunity against tularemia provided by an adenovirus-vectored vaccine expressing Tul4 of *Francisella tularensis*. Clin. Vaccine Immunol. 19, 359–364. 10.1128/CVI.05384-11 22278325 PMC3294612

[B65] Kim T. H. Pinkham J. T. Heninger S. J. Chalabaev S. Kasper D. L. (2012). Genetic modification of the O-polysaccharide of *Francisella tularensis* results in an avirulent live attenuated vaccine. J. Infect. Dis. 205, 1056–1065. 10.1093/infdis/jir620 21969334 PMC3295600

[B66] Kingry L. C. Petersen J. M. (2014). Comparative review of *Francisella tularensis* and Francisella novicida. Front. Cell. Infect. Microbiol. 4:35. 10.3389/fcimb.2014.00035 24660164 PMC3952080

[B67] Kostiala A. A. McGregor D. D. Logie P. S. (1975). Tularaemia in the rat. I. The cellular basis on host resistance to infection. Immunology 28, 855–69. 236983 PMC1445928

[B68] Lenco J. Pavkova I. Hubalek M. Stulik J. (2005). Insights into the oxidative stress response in *Francisella tularensis* LVS and its mutant DeltaiglC1+2 by proteomics analysis. FEMS Microbiol. Lett. 246, 47–54. 10.1016/j.femsle.2005.03.040 15869961

[B69] Li J. Ryder C. Mandal M. Ahmed F. Azadi P. Snyder D. S. (2007). Attenuation and protective efficacy of an O-antigen-deficient mutant of *Francisella tularensis* LVS. Microbiology 53, 3141–3153. 10.1099/mic.0.2007/006460-0 17768257

[B70] Lyons R. C. Wu T. H. (2007). Animal models of *Francisella tularensis* infection. Ann. N. Y. Acad. Sci. 1105, 238–265. 10.1196/annals.1409.003 17395735

[B71] Ma Z. Banik S. Rane H. Mora V. T. Rabadi S. M. Doyle C. R.. (2014). EmrA1 membrane fusion protein of *Francisella tularensis* LVS is required for resistance to oxidative stress, intramacrophage survival and virulence in mice. Mol. Microbiol. 91, 976–995. 10.1111/mmi.12509 24397487 PMC4097035

[B72] Mahawar M. Rabadi S. M. Banik S. Catlett S. V. Metzger D. W. Malik M.. (2013). Identification of a live attenuated vaccine candidate for tularemia prophylaxis. PLoS ONE 8:e61539. 10.1371/journal.pone.0061539 23613871 PMC3629233

[B73] Marohn M. E. Barry E. M. (2013). Live attenuated tularemia vaccines: recent developments and future goals. Vaccine 31, 3485–3491. 10.1016/j.vaccine.2013.05.096 23764535 PMC3766925

[B74] Matyas B. T. Nieder H. S. Telford S. R. 3rd (2007). Pneumonic tularemia on Martha's Vineyard: clinical, epidemiologic, and ecological characteristics. Ann. N. Y. Acad. Sci. 1105, 351–377. 10.1196/annals.1409.013 17442781

[B75] McCoy GW C. C. (1912). Further observations on a plaguelike disease of rodents with a preliminary note on the causative agent Bacterium tularense. J. Infect. Dis. 10, 61–72. 10.1093/infdis/10.1.61

[B76] McCrumb F. R. (1961). Aerosol infection of man with pasteurella tularensis. Bacteriol. Rev. 25, 262–267. 16350172 10.1128/br.25.3.262-267.1961 PMC441102

[B77] Meibom K. L. Dubail I. Dupuis M. Barel M. Lenco J. Stulik J.. (2008). The heat-shock protein ClpB of *Francisella tularensis* is involved in stress tolerance and is required for multiplication in target organs of infected mice. Mol. Microbiol. 67, 1384–401. 10.1111/j.1365-2958.2008.06139.x 18284578

[B78] Melillo A. A. Mahawar M. Sellati T. J. Malik M. Metzger D. W. Melendez J. A.. (2009). Identification of *Francisella tularensis* live vaccine strain CuZn superoxide dismutase as critical for resistance to extracellularly generated reactive oxygen species. J. Bacteriol. 191, 6447–6456. 10.1128/JB.00534-09 19684141 PMC2753026

[B79] Metzger D. W. Bakshi C. S. Kirimanjeswara G. (2007). Mucosal immunopathogenesis of *Francisella tularensis*. Ann. N. Y. Acad. Sci. 1105, 266–283. 10.1196/annals.1409.007 17395728

[B80] Michell S. L. Dean R. E. Eyles J. E. Hartley M. G. Waters E. Prior J. L.. (2010). Deletion of the Bacillus anthracis capB homologue in *Francisella tularensis* subspecies tularensis generates an attenuated strain that protects mice against virulent tularaemia. J. Med. Microbiol. 59, 1275–1284. 10.1099/jmm.0.018911-0 20651039

[B81] Mohapatra N. P. Soni S. Bell B. L. Warren R. Ernst R. K. Muszynski A.. (2007). Identification of an orphan response regulator required for the virulence of *Francisella* spp. and transcription of pathogenicity island genes. Infect. Immun. 75, 3305–3314. 10.1128/IAI.00351-07 17452468 PMC1932945

[B82] Mulligan M. J. Stapleton J. T. Keitel W. A. Frey S. E. Chen W. H. Rouphael N.. (2017). Tularemia vaccine: safety, reactogenicity, “Take” skin reactions, and antibody responses following vaccination with a new lot of the *Francisella tularensis* live vaccine strain - A phase 2 randomized clinical Trial. Vaccine 35, 4730–4737. 10.1016/j.vaccine.2017.07.024 28750854 PMC5800773

[B83] Nguyen J. Q. Zogaj X. Adelani A. A. Chu P. Yu J. J. Arulanandam B. P. (2017). Intratracheal inoculation of fischer 344 rats with *Francisella tularensis*. J. Vis. Exp. 127:e56123 10.3791/56123 PMC5752353 28994770

[B84] Pammit M. A. Raulie E. K. Lauriano C. M. Klose K. E. Arulanandam B. P. (2006). Intranasal vaccination with a defined attenuated Francisella novicida strain induces gamma interferon-dependent antibody-mediated protection against tularemia. Infect. Immun. 74, 2063–2071. 10.1128/IAI.74.4.2063-2071.2006 16552035 PMC1418901

[B85] Pasetti M. F. Cuberos L. Horn T. L. Shearer J. D. Matthews S. J. House R. V.. (2008). An improved *Francisella tularensis* live vaccine strain (LVS) is well tolerated and highly immunogenic when administered to rabbits in escalating doses using various immunization routes. Vaccine 26, 1773–1785. 10.1016/j.vaccine.2008.01.005 18308432 PMC2678717

[B86] Pavot V. Rochereau N. Genin C. Verrier B. Paul S. (2012). New insights in mucosal vaccine development. Vaccine 30, 142–154. 10.1016/j.vaccine.2011.11.003 22085556

[B87] Pechous R. Celli J. Penoske R. Hayes S. F. Frank D. W. Zahrt T. C. (2006). Construction and characterization of an attenuated purine auxotroph in a *Francisella tularensis* live vaccine strain. Infect. Immun. 74, 4452–4461. 10.1128/IAI.00666-06 16861631 PMC1539594

[B88] Pechous R. D. McCarthy T. R. Mohapatra N. P. Soni S. Penoske R. M. Salzman N. H.. (2008). A *Francisella tularensis* Schu S4 purine auxotroph is highly attenuated in mice but offers limited protection against homologous intranasal challenge. PLoS ONE 3:e2487. 10.1371/journal.pone.0002487 18575611 PMC2429968

[B89] Pechous R. D. McCarthy T. R. Zahrt T. C. (2009). Working toward the future: insights into *Francisella tularensis* pathogenesis and vaccine development. Microbiol. Mol. Biol. Rev. 73, 684–711. 10.1128/MMBR.00028-09 19946137 PMC2786580

[B90] Qin A. Scott D. W. Mann B. J. (2008). *Francisella tularensis* subsp. tularensis Schu S4 disulfide bond formation protein B, but not an RND-type efflux pump, is required for virulence. Infect. Immun. 76, 3086–3092. 10.1128/IAI.00363-08 18458069 PMC2446700

[B91] Qin A. Scott D. W. Rabideau M. M. Moore E. A. Mann B. J. (2011). Requirement of the CXXC motif of novel Francisella infectivity potentiator protein B FipB, and FipA in virulence of *F. tularensis* subsp. tularensis. PLoS ONE 6:e24611. 10.1371/journal.pone.0024611 21931773 PMC3169626

[B92] Qin A. Scott D. W. Thompson J. A. Mann B. J. (2009). Identification of an essential *Francisella tularensis* subsp. tularensis virulence factor. Infect. Immun. 77, 152–61. 10.1128/IAI.01113-08 18981253 PMC2612291

[B93] Quarry J. E. Isherwood K. E. Michell S. L. Diaper H. Titball R. W. Oyston P. C. (2007). A *Francisella tularensis* subspecies novicida purF mutant, but not a purA mutant, induces protective immunity to tularemia in mice. Vaccine 25, 2011–2018. 10.1016/j.vaccine.2006.11.054 17241711

[B94] Ray H. J. Chu P. Wu T. H. Lyons C. R. Murthy A. K. Guentzel M. N.. (2010). The Fischer 344 rat reflects human susceptibility to francisella pulmonary challenge and provides a new platform for virulence and protection studies. PLoS ONE 5:e9952. 10.1371/journal.pone.0009952 20376351 PMC2848594

[B95] Raymond C. R. Conlan J. W. (2009). Differential susceptibility of Sprague-Dawley and fischer 344 rats to infection by *Francisella tularensis*. Microb. Pathog. 46, 231–234. 10.1016/j.micpath.2009.01.002 19490832

[B96] Reed D. S. Smith L. P. Cole K. S. Santiago A. E. Mann B. J. Barry E. M. (2014). Live attenuated mutants of *Francisella tularensis* protect rabbits against aerosol challenge with a virulent type A strain. Infect. Immun. 82, 2098–2105. 10.1128/IAI.01498-14 24614653 PMC3993426

[B97] Rockx-Brouwer D. Chong A. Wehrly T. D. Child R. Crane D. D. Celli J.. (2012). Low dose vaccination with attenuated *Francisella tularensis* strain SchuS4 mutants protects against tularemia independent of the route of vaccination. PLoS ONE 7:e37752. 10.1371/journal.pone.0037752 22662210 PMC3360632

[B98] Rohmer L. Fong C. Abmayr S. Wasnick M. Larson Freeman T. J. Radey M.. (2007). Comparison of *Francisella tularensis* genomes reveals evolutionary events associated with the emergence of human pathogenic strains. Genome Biol. 8:R102. 10.1186/gb-2007-8-6-r102 17550600 PMC2394750

[B99] Ryden P. Twine S. Shen H. Harris G. Chen W. Sjostedt A.. (2013). Correlates of protection following vaccination of mice with gene deletion mutants of *Francisella tularensis* subspecies tularensis strain, SCHU S4 that elicit varying degrees of immunity to systemic and respiratory challenge with wild-type bacteria. Mol. Immunol. 54, 58–67. 10.1016/j.molimm.2012.10.043 23201853

[B100] Saha S. S. Hashino M. Suzuki J. Uda A. Watanabe K. Shimizu T.. (2017). Contribution of methionine sulfoxide reductase B (MsrB) to *Francisella tularensis* infection in mice. FEMS Microbiol. Lett. 364:fnw260. 10.1093/femsle/fnw260 28108583

[B101] Salomonsson E. Kuoppa K. Forslund A. L. Zingmark C. Golovliov I. Sjostedt A.. (2009). Reintroduction of two deleted virulence loci restores full virulence to the live vaccine strain of *Francisella tularensis*. Infect. Immun. 77, 3424–3431. 10.1128/IAI.00196-09 19506014 PMC2715654

[B102] Sammons-Jackson W. L. McClelland K. Manch-Citron J. N. Metzger D. W. Bakshi C. S. Garcia E.. (2008). Generation and characterization of an attenuated mutant in a response regulator gene of *Francisella tularensis* live vaccine strain (LVS). DNA Cell Biol. 27, 387–403. 10.1089/dna.2007.0687 18613792 PMC2980773

[B103] Sanapala S. Yu J. J. Murthy A. K. Li W. Guentzel M. N. Chambers J. P.. (2012). Perforin- and granzyme-mediated cytotoxic effector functions are essential for protection against *Francisella tularensis* following vaccination by the defined F. tularensis subsp. novicida DeltafopC vaccine strain. Infect. Immun. 80, 2177–2185. 10.1128/IAI.00036-12 22493083 PMC3370591

[B104] Santiago A. E. Cole L. E. Franco A. Vogel S. N. Levine M. M. Barry E. M. (2009). Characterization of rationally attenuated *Francisella tularensis* vaccine strains that harbor deletions in the guaA and guaB genes. Vaccine 27, 2426–2436. 10.1016/j.vaccine.2009.02.073 19368784 PMC2716139

[B105] Santiago A. E. Mann B. J. Qin A. Cunningham A. L. Cole L. E. Grassel C.. (2015). Characterization of *Francisella tularensis* Schu S4 defined mutants as live-attenuated vaccine candidates. Pathogens Dis. 73:ftv036. 10.1093/femspd/ftv036 25986219 PMC4462183

[B106] Saslaw S. Carhart S. (1961). Studies with tularemia vaccines in volunteers. III. Serologic aspects following intracutaneous or respiratory challenge in both vaccinated and nonvaccinated volunteers. Am. J. Med. Sci. 241, 689–699. 13746662

[B107] Saslaw S. Eigelsbach H. T. Prior J. A. Wilson H. E. Carhart S. (1961a). Tularemia vaccine study. II. Respiratory challenge. Arch. Intern. Med. 107, 702–714. 10.1001/archinte.1961.03620050068007 13746667

[B108] Saslaw S. Eigelsbach H. T. Wilson H. E. Prior J. A. Carhart S. (1961b). Tularemia vaccine study. I. Intracutaneous challenge. Arch. Intern. Med. 107, 689–701. 10.1001/archinte.1961.03620050055006 13746668

[B109] Schmitt D. M. O'Dee D. M. Horzempa J. Carlson P. E. Jr. Russo B. C. Bales J. M.. (2012). A *Francisella tularensis* live vaccine strain that improves stimulation of antigen-presenting cells does not enhance vaccine efficacy. PLoS ONE 7:e31172. 10.1371/journal.pone.0031172 22355343 PMC3280287

[B110] Schricker R. L. Eigelsbach H. T. Mitten J. Q. Hall W. C. (1972). Pathogenesis of tularemia in monkeys aerogenically exposed to *Francisella tularensis* 425. Infect. Immun. 5, 734–744. 4629251 10.1128/iai.5.5.734-744.1972 PMC422433

[B111] Sebastian S. Dillon S. T. Lynch J. G. Blalock L. T. Balon E. Lee K. T.. (2007). A defined O-antigen polysaccharide mutant of *Francisella tularensis* live vaccine strain has attenuated virulence while retaining its protective capacity. Infect. Immun. 75, 2591–602. 10.1128/IAI.01789-06 17296751 PMC1865767

[B112] Sebastian S. Pinkham J. T. Lynch J. G. Ross R. A. Reinap B. Blalock L. T.. (2009). Cellular and humoral immunity are synergistic in protection against types A and B *Francisella tularensis*. Vaccine 27, 597–605. 10.1016/j.vaccine.2008.10.079 19022323 PMC3005699

[B113] Shen H. Chen W. Conlan J. W. (2004). Mice sublethally infected with Francisella novicida U112 develop only marginal protective immunity against systemic or aerosol challenge with virulent type A or B strains of *F. tularensis*. Microb. Pathog. 37, 107–110. 10.1016/j.micpath.2004.04.005 15312850

[B114] Shen H. Harris G. Chen W. Sjostedt A. Ryden P. Conlan W. (2010). Molecular immune responses to aerosol challenge with *Francisella tularensis* in mice inoculated with live vaccine candidates of varying efficacy. PLoS ONE 5:e13349. 10.1371/journal.pone.0013349 20967278 PMC2953512

[B115] Signarovitz A. L. Ray H. J. Yu J. J. Guentzel M. N. Chambers J. P. Klose K. E.. (2012). Mucosal immunization with live attenuated Francisella novicida U112DeltaiglB protects against pulmonary F. tularensis SCHU S4 in the Fischer 344 rat model. PLoS ONE 7:e47639. 10.1371/journal.pone.0047639 23118885 PMC3484155

[B116] Sjostedt A. (2007). Tularemia: history, epidemiology, pathogen physiology, and clinical manifestations. Ann. N. Y. Acad. Sci. 1105, 1–29. 10.1196/annals.1409.009 17395726

[B117] Sjostedt A. Eriksson M. Sandstrom G. Tarnvik A. (1992a). Various membrane proteins of *Francisella tularensis* induce interferon-gamma production in both CD4+ and CD8+ T cells of primed humans. Immunology 76, 584–592. 1356911 PMC1421560

[B118] Sjostedt A. Sandstrom G. Tarnvik A. (1992b). Humoral and cell-mediated immunity in mice to a 17-kilodalton lipoprotein of *Francisella tularensis* expressed by *Salmonella typhimurium*. Infect. Immun. 60, 2855–2862. 1612751 10.1128/iai.60.7.2855-2862.1992 PMC257245

[B119] Straskova A. Spidlova P. Mou S. Worsham P. Putzova D. Pavkova I. (2015). Francisella tularensis type B DeltadsbA mutant protects against type A strain and induces strong inflammatory cytokine and Th1-like antibody response *in vivo. Pathogens Dis* 73:ftv058. 10.1093/femspd/ftv058 PMC4626617 26253078

[B120] Su J. Asare R. Yang J. Nair M. K. Mazurkiewicz J. E. Abu-Kwaik Y.. (2011). The capBCA locus is required for intracellular growth of *Francisella tularensis* LVS. Front. Microbiol. 2:83. 10.3389/fmicb.2011.00083 21747799 PMC3128946

[B121] Su J. Yang J. Zhao D. Kawula T. H. Banas J. A. Zhang J. R. (2007). Genome-wide identification of *Francisella tularensis* virulence determinants. Infect. Immun. 75, 3089–3101. 10.1128/IAI.01865-06 17420240 PMC1932872

[B122] Suresh R. V. Ma Z. Sunagar R. Bhatty V. Banik S. Catlett S. V.. (2015). Preclinical testing of a vaccine candidate against tularemia. PLoS ONE 10:e0124326. 10.1371/journal.pone.0124326 25897786 PMC4405390

[B123] Tarnvik A. (1989). Nature of protective immunity to *Francisella tularensis*. Rev. Infect. Dis. 11, 440–451. 10.1093/clinids/11.3.440 2665002

[B124] Tempel R. Lai X. H. Crosa L. Kozlowicz B. Heffron F. (2006). Attenuated Francisella novicida transposon mutants protect mice against wild-type challenge. Infect. Immun. 74, 5095–5105. 10.1128/IAI.00598-06 16926401 PMC1594869

[B125] Twine S. Bystrom M. Chen W. Forsman M. Golovliov I. Johansson A.. (2005). A mutant of *Francisella tularensis* strain SCHU S4 lacking the ability to express a 58-kilodalton protein is attenuated for virulence and is an effective live vaccine. Infect. Immun. 73, 8345–8352. 10.1128/IAI.73.12.8345-8352.2005 16299332 PMC1307091

[B126] Twine S. Shen H. Harris G. Chen W. Sjostedt A. Ryden P. (2012). BALB/c mice, but not C57BL/6 mice immunized with a DeltaclpB mutant of *Francisella tularensis* subspecies tularensis are protected against respiratory challenge with wild-type bacteria: association of protection with post-vaccination and post-challenge immune responses. Vaccine 30, 3634–3645. 10.1016/j.vaccine.2012.03.036 22484348

[B127] Vanmetre T. E. Kadull P. J. (1959). Laboratory-acquired tularemia in vaccinated individuals - a report of 62 Cases. Ann. Intern. Med. 50, 621–632. 10.7326/0003-4819-50-3-621 13627717

[B128] Wayne Conlan J. Oyston P. C. (2007). Vaccines against *Francisella tularensis*. Ann. N. Y. Acad. Sci. 1105, 325–350. 10.1196/annals.1409.012 17395730

[B129] Weinberg A. N. (2004). Commentary: Wherry WB, Lamb BH. Infection of man with Bacterium tularense. J Infect Dis 15, 331-40. J. Infect. Dis. 189, 1317–1320. 10.1086/382546 15031803

[B130] Weiss D. S. Brotcke A. Henry T. Margolis J. J. Chan K. Monack D. M. (2007). In vivo negative selection screen identifies genes required for *Francisella virulence*. Proc. Natl. Acad. Sci. U.S.A. 104, 6037–6042. 10.1073/pnas.0609675104 17389372 PMC1832217

[B131] West T. E. Pelletier M. R. Majure M. C. Lembo A. Hajjar A. M. Skerrett S. J. (2008). Inhalation of *Francisella novicida* Delta mglA causes replicative infection that elicits innate and adaptive responses but is not protective against invasive pneumonic tularemia. Microbes Infect. 10, 773–780. 10.1016/j.micinf.2008.04.008 18539500 PMC2657097

[B132] WHO (2007). WHO Guidelines on Tularaemia. WHO Press, World Health Organization, Switzerland Available online at: https://www.cdc.gov/tularemia/resources/whotularemiamanual.pdf

[B133] Wu T. H. Hutt J. A. Garrison K. A. Berliba L. S. Zhou Y. Lyons C. R. (2005). Intranasal vaccination induces protective immunity against intranasal infection with virulent *Francisella tularensis* biovar A. Infect. Immun. 73, 2644–2654. 10.1128/IAI.73.5.2644-2654.2005 15845466 PMC1087315

[B134] Wu T. H. Zsemlye J. L. Statom G. L. Hutt J. A. Schrader R. M. Scrymgeour A. A.. (2009). Vaccination of Fischer 344 rats against pulmonary infections by *Francisella tularensis* type A strains. Vaccine 27, 4684–4693. 10.1016/j.vaccine.2009.05.060 19520198 PMC2732339

[B135] Zarrella T. M. Singh A. Bitsaktsis C. Rahman T. Sahay B. Feustel P. J.. (2011). Host-adaptation of *Francisella tularensis* alters the bacterium's surface-carbohydrates to hinder effectors of innate and adaptive immunity. PLoS ONE 6:e22335. 10.1371/journal.pone.0022335 21799828 PMC3142145

[B136] Zhou B. Meliopoulos V. A. Wang W. Lin X. Stucker K. M. Halpin R. A.. (2016). Reversion of cold-adapted live attenuated influenza vaccine into a pathogenic virus. J. Virol. 90, 8454–8463. 10.1128/JVI.00163-16 27440882 PMC5021423

